# Band Gap Engineering and Trap Depths of Intrinsic Point Defects in RAlO_3_ (R = Y, La, Gd, Yb, Lu) Perovskites

**DOI:** 10.1021/acs.jpcc.1c06573

**Published:** 2021-11-23

**Authors:** Yaroslav Zhydachevskyy, Yuriy Hizhnyi, Sergii G. Nedilko, Irina Kudryavtseva, Vladimir Pankratov, Vasyl Stasiv, Leonid Vasylechko, Dmytro Sugak, Aleksandr Lushchik, Marek Berkowski, Andrzej Suchocki, Nickolai Klyui

**Affiliations:** † Institute of Physics, Polish Academy of Sciences, aleja Lotników 32/46, Warsaw 02-668, Poland; ‡ Lviv Polytechnic National University, S. Bandera Str. 12, Lviv 79013, Ukraine; § Taras Shevchenko National University of Kyiv, Volodymyrska Str. 60, Kyiv 01033, Ukraine; ∥ Institute of Physics, University of Tartu, W. Ostwald Str. 1, Tartu 50411, Estonia; ⊥ Institute of Solid State Physics, University of Latvia, Kengaraga Str. 8, Riga 1063, Latvia; # College of Physics, Jilin University, 2699 Qianjin Str., Changchun 130012, China; ∇ V.E. Lashkaryov Institute of Semiconductor Physics, National Academy of Sciences of Ukraine, 41 prospekt Nauki, Kyiv 03028, Ukraine

## Abstract

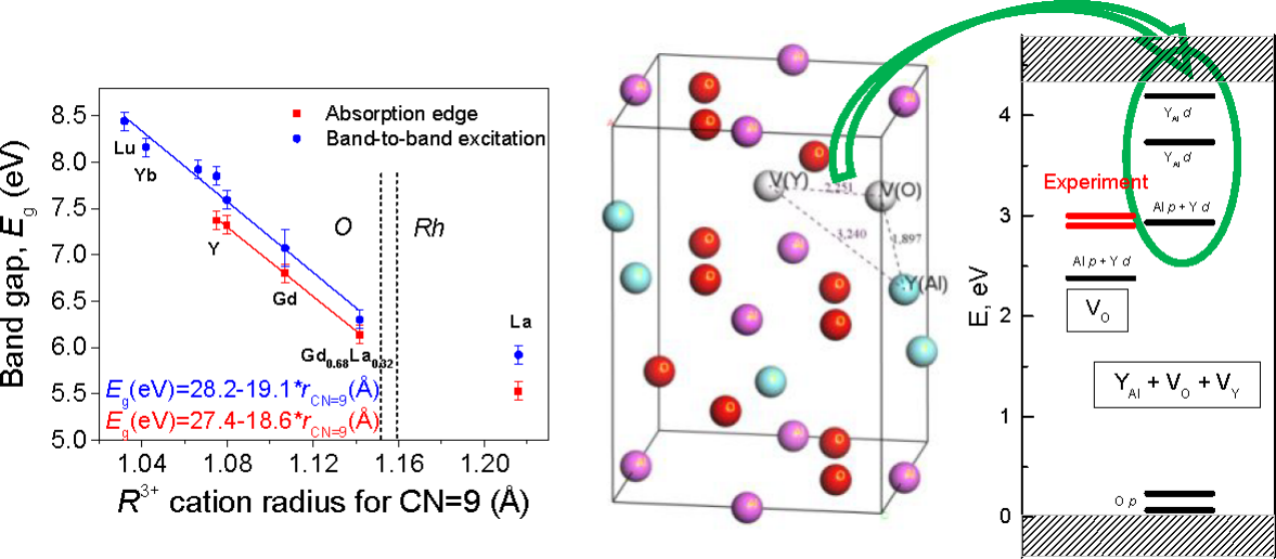

The possibility of band gap engineering (BGE) in RAlO_3_ (R = Y, La, Gd, Yb, Lu) perovskites in the context of trap depths of intrinsic point defects was investigated comprehensively using experimental and theoretical approaches. The optical band gap of the materials, *E*_g_, was determined via both the absorption measurements in the VUV spectral range and the spectra of recombination luminescence excitation by synchrotron radiation. The experimentally observed effect of *E*_g_ reduction from ∼8.5 to ∼5.5 eV in RAlO_3_ perovskites with increasing R^3+^ ionic radius was confirmed by the DFT electronic structure calculations performed for RM^III^O_3_ crystals (R = Lu, Y, La; M^III^ = Al, Ga, In). The possibility of BGE was also proved by the analysis of thermally stimulated luminescence (TSL) measured above room temperature for the far-red emitting (Y/Gd/La)AlO_3_:Mn^4+^ phosphors, which confirmed decreasing of the trap depths in the cation sequence Y → Gd → La. Calculations of the trap depths performed within the super cell approach for a number of intrinsic point defects and their complexes allowed recognizing specific trapping centers that can be responsible for the observed TSL. In particular, the electron traps of 1.33 and 1.43 eV (in YAlO_3_) were considered to be formed by the energy level of oxygen vacancy (V_O_) with different arrangement of neighboring Y_Al_ and V_Y_, while shallower electron traps of 0.9–1.0 eV were related to the energy level of Y_Al_ antisite complexes with neighboring V_O_ or (V_O_ + V_Y_). The effect of the lowering of electron trap depths in RAlO_3_ was demonstrated for the V_O_-related level of the (Y_Al_ + V_O_ + V_Y_) complex defect for the particular case of La substituting Y.

## 1 Introduction

**Figure 1:**
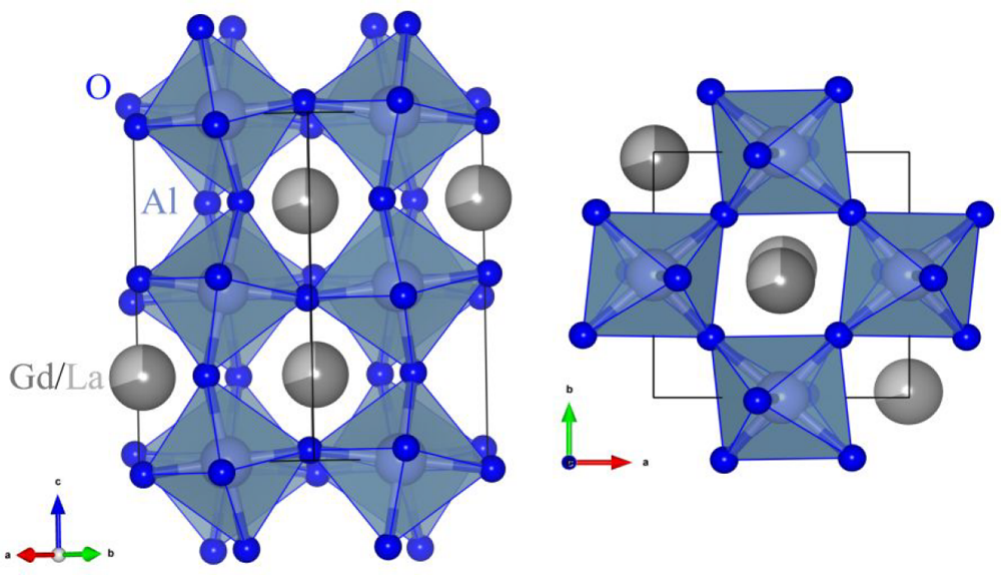
Visualization of the RAlO_3_ (R = Y, La, Gd, Yb, Lu) unit cell using AlO_6_ octahedra.

Yttrium–aluminum perovskite (YAlO_3_ or YAP) is a well-known host material for solid-state lasers, scintillators, and various kinds of converting and storage phosphors (see, e.g., refs (1−6) and references therein). YAlO_3_ crystal possesses deformed perovskite GdFeO_3_ type structure with orthorhombic symmetry (space group *D*_2*h*_^16^–*Pbnm*).^7^ The structure can be represented by a network of slightly distorted and turned AlO_6_ octahedra connected by apexes, where Y^3+^ ions are located inside, thus forming strongly distorted YO_12_ cavities with the nearest 8 or 9 oxygen ions around Y^3+^ (see Figure 1). Other rare-earth-based aluminates and their solid solutions with the same type of structure, such as LuAlO_3_, Y_1–*x*_Lu_*x*_AlO_3_, GdAlO_3_, YbAlO_3_, *etc.*, are also well-known.^8−11^

A huge amount of important optical and luminescent properties of these materials is influenced by the energy levels formed inside the forbidden gap of the material by activator ions, native point defects, and uncontrolled impurities. The location of these energy levels relative to the edges of conduction or valence electronic bands is crucial for the radiation-induced and thermally induced processes, like ionization, charge trapping and storage, recombination, energy transfer, *etc.*

Band gap engineering (BGE) in complex oxide crystals is a concept of elimination of shallow-trap defect states in the crystals by cation substitution. It was first proposed for R_3_M^III^_5_O_12_ garnets by Fasoli *et al*.^12^ The concept is based on the assumption that substitution of M^III^ cations in such crystals can lead to the enveloping of some defect levels, initially located in their band gaps, by the band electronic states. Such deactivation of defect states can improve the scintillation characteristics of the doped crystals (or mixed-cationic solid solutions) relative to initial undoped ones. For instance, if Al cations are substituted with Ga in R_3_Al_5_O_12_ garnet crystal, some shallow defect levels in Al-garnet may be presumably enveloped by the conduction band states of Ga-garnet, because R_3_Ga_5_O_12_ crystals have narrower band gaps than corresponding R_3_Al_5_O_12_.^13^

It is known that the conduction band (CB) of a YAlO_3_ perovskite crystal is formed mainly by Y 4d states, whereas the valence band (VB) is formed by a superposition of O 2p and Al 3p states.^14^ Therefore, replacement of yttrium or aluminum in this kind of material by some other metal or rare-earth cation should affect the forbidden gap width. Applicability of the BGE approach in perovskites via variation of their composition has recently been confirmed experimentally. It has been shown that the variation of the Gd/La ratio in Gd_1–*x*_La_*x*_AlO_3_ perovskites doped with Eu^3+^/Pr^3+^ or Eu^3+^/Tb^3+^ is an efficient tool for tuning of the defect- or dopant-related trap depths.^15^

However, it is obvious that a key *postulate* has to be accepted to make the BGE concept valid: cation substitution should not only change the band gap but also decrease/increase the defect level position with respect to the band gap edges (which eventually results in enveloping the level by the band states). It is also obvious that the position of a shallow defect level in the band gap may be shifted as a result of cation substitution, thus keeping the energy depth of the corresponding charge carrier trap practically unchanged and thus eliminating the effect of BGE. The influence of cation substitution on a particular defect in a specific crystal is hardly predictable *a priori*.

Narrowing of the band gap width, *E*_g_, by cation substitution has obtained computational evidence for several cases of garnet compounds, like Lu_3_(Al_*x*_Ga_1–*x*_)_5_O_12_^12^ and Y_3_(Al_*x*_Ga_1–*x*_)_5_O_12_.^16^ However, to the best of our knowledge, there are no direct computational results demonstrating the changes of trap depths of specific defects in garnets or other oxide compounds.

There were also several computational efforts that considered the BGE problem in perovskites. Density functional theory (DFT)-based computational studies with use of defect-containing super cells were applied to LuAlO_3_.^17^ It was shown that in such a crystal, substitution of M^III^ cations from Al to Ga can lead to enveloping of the defect levels of some electron traps by the CB. In particular, the levels of Lu_i_, Lu_Ga_, and Ga_Lu_ defects fall into the conduction band of LuGaO_3_.^17^ However, calculations of the defect levels were done by Liu *et al*.^17^ only for LuAlO_3_ and LuGaO_3_ crystal hosts. It is obvious that more reliable results on the defect level behavior with cationic substitution can be obtained with the use of super cells of the solid solutions, like LuAl_*x*_Ga_1–*x*_O_3_.

In order to know the limits and possibilities of implementation of the BGE approach in the RAlO_3_ perovskites, it is necessary to understand the effect of the R cation substitution on the electronic structure and, in particular, on the *E*_g_ value. Only scarce information about this issue can be found in the literature. For example, from the luminescence studies under synchrotron radiation excitation, it is known that LuAlO_3_ has a band gap width at least 0.6 eV larger than YAlO_3_,^18,19^ whereas GdAlO_3_ probably has a narrower band gap than YAlO_3_.^20^ The replacement of Gd by La gradually decreases the *E*_g_ of Gd_1–*x*_La_*x*_AlO_3_.^15^ Therefore, there is an obvious lack of a systematic study demonstrating the effect of various rare-earth (R) cations on the band gap width of RAlO_3_ perovskites. This knowledge is very important for controllable tuning of the defect- or dopant-related trap depths and for improvement thereby of the performance of scintillator materials, storage, or persistent luminescence phosphors. The present work is aimed to eliminate this shortage. Dependence of the *E*_g_ of RAlO_3_ perovskites on the type of the R cation (R = Y, La, Gd, Yb, Lu) is determined in systematic theoretical and experimental studies.

The DFT-based theoretical calculations with the use of the plane-wave pseudopotential method have been carried out in order to establish the effect of R cation substitution on the electronic band structure and *E*_g_ value of RAlO_3_ crystals. Results obtained from the calculations are compared with the experimental estimations of *E*_g_ values for RAlO_3_ perovskites. In particular, optical absorption of the single crystals in the UV–VUV range and luminescence measurements of the same crystals under synchrotron radiation excitation were performed. In addition, the thermally stimulated luminescence (TSL) measurements of the Mn^4+^-doped microcrystalline Y_1–*x*_Gd_*x*_AlO_3_ and Gd_1–*y*_La_*y*_AlO_3_ phosphors have been performed in the temperature range from 300 to 500 °C in order to determine the effect of Gd and La doping on the trap depths formed by native defects in these materials.

While the effect of R cation substitution on the *E*_g_ of perovskites can be established in such systematic studies, the effect of such substitution on positions of the defect levels with respect to the band edges is a nontrivial problem and requires special studies for particular defects and hosts. In the present paper, we study this problem computationally by considering one meaningful example, namely, substitution of Y with La in YAlO_3_ crystal. The effect of La doping on the defect level positions in the band gap of YAlO_3_ was determined via calculations with the use of the DFT method within the super cell approach. A wide set of defects of different types has been considered in calculations in order to find such defect combinations that most probably determine the high-temperature TSL peaks of the synthesized perovskites crystals.

## 2 Experimental and Calculation Methods

### 2.1 Sample Preparation and Experimental Methods

Single-crystalline RAlO_3_ perovskite crystals studied in this work were grown by the Czochralski method in inert gas atmosphere at the Institute of Physics PAS, Institute of Electronic Materials Technology or Norfolk State University (see Acknowledgments). For the absorption and luminescence measurements, the samples were prepared as plane-parallel double-side polished plates of 50–100 μm thickness.

Beside the single crystals, two series of Mn^4+^-doped ceramic samples were specially prepared for the purposes of this work. Namely, Y_1–*x*_Gd_*x*_AlO_3_:Mn^4+^ (*x* = 0, 0.2, 0.4, 0.6, 0.8, 1.0) and Gd_1–*y*_La_*y*_AlO_3_:Mn^4+^ (*y* = 0, 0.2, 0.4) samples in the form of microcrystalline powders were synthesized by a conventional solid-state reaction in air atmosphere. For this purpose, starting materials of Y_2_O_3_, Gd_2_O_3_, La_2_O_3_, Al_2_O_3_, and MnO_2_ in the form of high-purity (not worse than 99.99%) microcrystalline powders were used. To prompt higher TSL response of the Mn^4+^-doped ceramic samples,^64^ they were purposely synthesized from the R-rich composition corresponding to the nominal chemical formula of R_1.02_Al_0.98_O_3_ (R = Y, Gd, La). After thorough mixing, the mixture was pressed into pellets 1/2 in. in diameter and calcined at temperature up to 1600 °C in three stages (36 h overall) with an intermediate grinding and pressing in between. After synthesis, the solid ceramic samples were ground again to get the fine powder that has been studied.

The optical absorbance spectra were measured using a spectrophotometer JASCO V-660 with a double monochromator (1.5–6.5 eV) and laboratory setup based on a vacuum monochromator VMR-2 and a hydrogen discharge light source (5.5–11 eV). In the latter case, the constant number of exciting photons was achieved by varying the slit width of the monochromator and using the constant signal from sodium salicylate for normalization.

The luminescence properties of the perovskite single crystals in the VUV spectral range were examined using synchrotron radiation. The luminescence experiments were carried out on the photoluminescence end station FINESTLUMI^21,22^ of the FinEstBeAMS beamline,^23,24^ at the 1.5 GeV storage ring of MAX IV synchrotron facility (Lund, Sweden). The excitation spectra of luminescence were normalized utilizing the calibration curve obtained by the AXUV-100G diode. Luminescence detection in the UV–visible–IR spectral range (200–850 nm) was performed by the Andor Shamrock (SR-303i) 0.3 m spectrometer having two gratings (300 grooves/mm and blaze @300 nm (300/300) or blaze @500 nm (300/500)). The Andor Shamrock spectrometer was equipped with photomultiplier photon counting heads (H8259-01 Hamamatsu) covering the spectral range from 200 to 900 nm. The perovskite single crystals were mounted on the sample holder of the close-cycle cryostat inserted into the UHV (10^–9^ mbar) chamber, and experiments were carried out at 10 K.

Phase and structural characterization of the materials prepared was performed by the X-ray powder diffraction (XRPD) technique. Experimental diffraction patterns were collected on the modernized X-ray powder diffractometer DRON-3 M in Cu Kα radiation (λ = 1.54185 Å) in the 2θ range of 15–120° and 2θ step of 0.02°. Structural parameters of the studied Y_1–*x*_Gd_*x*_AlO_3_:Mn^4+^ and Gd_1–*y*_La_*y*_AlO_3_:Mn^4+^ samples were derived from the experimental XRPD data by full profile Rietveld refinement using the WinCSD software package.^25^ In the refinement procedure, lattice parameters, coordinates, and displacement parameters of atoms of the main perovskite phase were refined together with profile parameters and corrections for absorption and instrumental sample shift. Simultaneous multiphase Rietveld refinement was also used for a quantitative phase analysis of the materials synthesized.

TSL measurements of the Mn^4+^-doped microcrystalline phosphors were done above room temperature using a laboratory TL-reader with a Hamamatsu R928 photomultiplier. A red long-pass filter (cutting off <650 nm) was used in the TSL measurements. To estimate an activation energy from the thermal glow (TSL) curves, the initial rise method and the partial cleaning procedure were used.

### 2.2 Theoretical Calculations

The geometry-optimized electronic structure calculations were carried out in spin-polarized mode using the DFT-based plane-wave pseudopotential method implemented in CASTEP^26^ package of commercial program pack.^27^ The ion–electron interactions were modeled by Vanderbilt-type nonlocal ultrasoft pseudopotentials.^28^ The following orbital electrons were regarded as valence electrons: Y 4d^1^5s^2^, La 5s^2^5p^6^5d^1^6s^2^, Gd 5s^2^6s^2^5p^6^5d^1^4f^7^, Yb 4f^14^5s^2^5p^6^6s^2^, Lu 4f^14^5p^6^5d^1^6s^2^, Al 3s^2^3p^1^, Ga 3d^10^4s^2^4p^1^, In 4d^10^5s^2^5p^1^, Si 3s^2^3p^2^, Hf 5d^2^6s^2^, and O 2s^2^2p^4^. The optimization was carried out by the Broyden–Fletcher–Goldfarb–Shanno (BFGS) minimization technique,^29^ using the following convergence criteria: an energy tolerance of 10^–5^ eV/atom; the maximum Hellman–Feynman forces of 0.03 eV/Å; and maximum stress and maximum displacement of 0.05 GPa and 10^–3^ Å, respectively.

The electronic structures of perfect RAlO_3_ (R = Y, La, Gd, Yb, Lu) perovskite crystals were calculated with various exchange–correlation functionals and related approximations:^27^ GGA-PBE, PBE0, HSE03, B3LYP, and several others for perfect YAlO_3_ (the list of functionals used for a particular crystal is presented in the Supporting Information, Table S1). The LDA+U method was used for the corrections of on-site Coulomb interactions with *U* = 6 and 20 eV for Gd f and Yb f states, respectively.^30,31^ The ensemble density functional theory (EDFT)^32^ scheme was used to overcome the convergence problem of the systems with f-states.

The crystal structure parameters of perfect RAlO_3_ crystals were taken from literature data^33−38^ (see also Table S1). Optimizations of the unit cell parameters and fractional atomic coordinates have led to slight increase of the unit cell volumes of all crystals (within ∼1% of the absolute values). Such changes of the crystal volumes are typical for the calculation method and exchange–correlation functionals applied here, and their detailed analysis is beyond the scope of this paper.

The energy cutoff of the plane-wave basis set was 340.0 eV, and a Monkhorst–Pack mesh of 5 × 5 × 5 k-points in the Brillouin zone was used in calculations for the perfect RAlO_3_ crystals. The partial densities of states (PDOS) were calculated by summing up spin-up and spin-down cases and using Gaussian broadening with 0.1 eV smearing width. Additional convergence tests showed that the choice of the above computational parameters is sufficiently accurate in this study. It was also checked that using a finer k-point mesh leads to very weak redistributions of the PDOS pictures.

Geometry-optimized calculations of the electronic structure of YAlO_3_ crystals with defects were performed in the super cell approach. The super cells were constructed as 2 × 2 × 2 multiplication of the unit cell and comprised 160 atoms of the crystal. The symmetries of super cells were set to primitive group *P*1. Calculations for the defect-containing super cells were done for the Γ point of the Brillouin zone with use of GGA-PBE approximation of exchange–correlation potential. Other approximations and parameters of computational procedure were the same as in the case of perfect RAlO_3_ crystals (see above).

Several kinds of point defects and defect combinations were modeled in the super cells of YAlO_3_: natural vacancies (V_O_, V_Y_, V_Al_) and vacancy complexes (V_O_ + V_Y_) and (V_O_ + V_Al_), interstitial oxygen defects O_i_, and combinations of such interstitials with natural vacancies (V_O_ + O_i_) and (V_Y_ + O_i_), cationic antisites (Y_Al_, Al_Y_), iso- (La_Y_, La_Al_, Ga_Y_) and aliovalent cationic substitutions (Hf_Y_, Si_Al_), as well as several other combinations of such defects (Y_Al_ + V_O_), (Y_Al_ + V_Y_), (Y_Al_ + V_Al_), (Y_Al_ + O_i_), (Y_Al_ + Al_Y_), (2Y_Al_ + V_O_), (Y_Al_ + V_O_ + O_i_), (Y_Al_ + V_O_ + V_Y_), (V_O_ + V_Y_ + 2Y_Al_), including (Y_Al_ + V_O_ + V_Y_) defect in Y_0.75_La_0.25_AlO_3_ mixed-cationic solid solution. In the super cell of the latter defect, eight nearest-neighboring to Y_Al_ octahedral cationic positions of YAlO_3_ lattice were filled by lanthanum atoms (La_Y_ substitutions), while the remaining 24 octahedral positions were occupied by Y, thus providing the Y_0.75_La_0.25_AlO_3_ chemical formula for the crystal. Corresponding structural *.cif files of geometry optimized for perfect and defect super cells of the YAlO_3_ crystal are provided in the Supporting Information as an archive file (cifs.zip). The defects were modeled by removing (adding) the neutral atoms from the super cells without imposing any additional charge to the system. The choice of the above-described set of defects is substantiated in 3.4 Theoretical Calculations.

It should be noted that at the used super cell size (10.4 × 10.7 × 14.7 Å^3^), the distance between defects in the nearest super cells is not larger than ∼7 Å, whereas the longest interatomic distance in the most spatially extensive complex defect modeled here equals 3.3 Å, which is approximately twice smaller. Thereofore, there is a good reason to assume in calculations that the mutual influence of defects from the neighboring super cells is negligible in calculations.

Positions of the defect levels with respect to the band edges of YAlO_3_ crystal were derived from a thorough comparison between PDOS distributions of the perfect and defect-containing super cells calculated with smearing width 0.01 eV.

## 3 Results and Discussion

### 3.1 VUV Absorption Measurements

A commonly used technique for determination of the optical band gaps of crystals (in some approximations, the optical band gap characterizes the *E*_g_ value of a crystal, but usually underestimates it) is a construction of the Tauc plots using the experimentally measured optical absorbance spectra (see, e.g. Zatovsky *et al*.^39^ and references therein). In this technique, the optical absorption spectra α(*hν*) are used for the construction of (α*hν*)^1/*n*^ dependencies on *hν*. The crossing points are then taken as the optical band gap values. The choice of *n* parameter depends on the origin of electronic transitions. For crystals, it should be taken as *n* = 1/2, if the band-to-band transitions are direct, or as *n* = 2 if the interband transitions are indirect. Then the linear regions in these dependencies are extrapolated until crossing the abscissa.

As our calculations of *E*(*k*) curves show (band dispersion curves calculated for two spin directions α or β and partial densities of states are presented in the Supporting Information, in Figures S1 and S2, respectively, and corresponding band gap parameters are listed in Table S2), some of the studied RM^III^O_3_ crystals are indirect-band gap materials (YAlO_3_, LaAlO_3_, and YInO_3_), whereas all others have direct band gaps. For this reason, we estimate the optical band gaps of YAlO_3_, LaAlO_3_, and YInO_3_ from Tauc plots constructed with *n* = 2 and use *n* = 1/2 for all other crystals.

**Figure 2:**
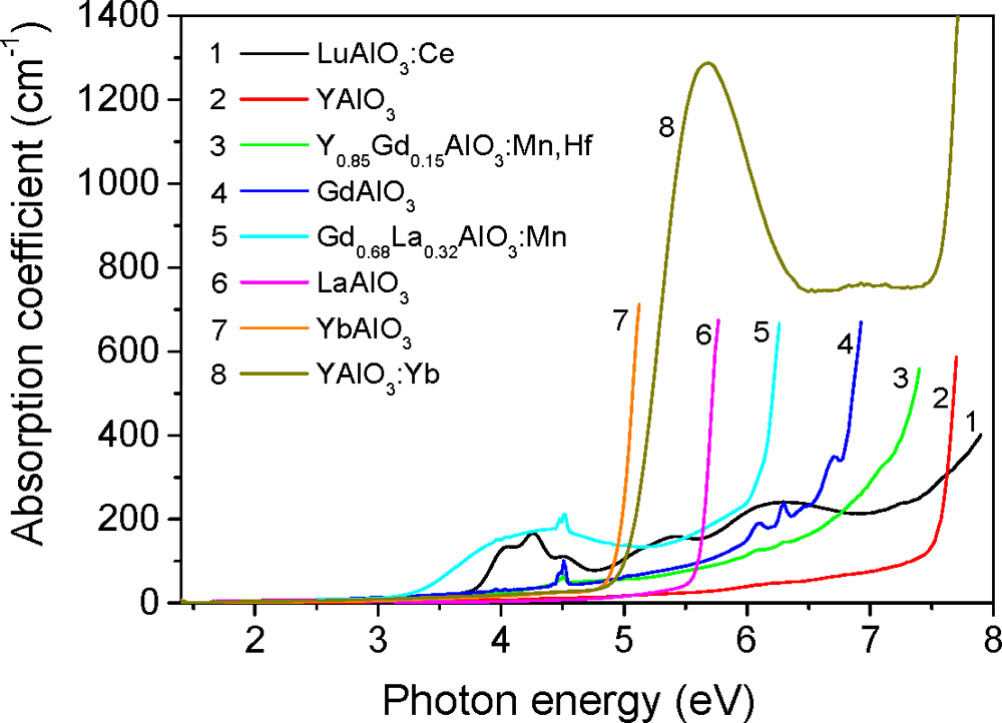
Absorption spectra of single-crystalline RAlO_3_ compounds measured at room temperature.

**Figure 3:**
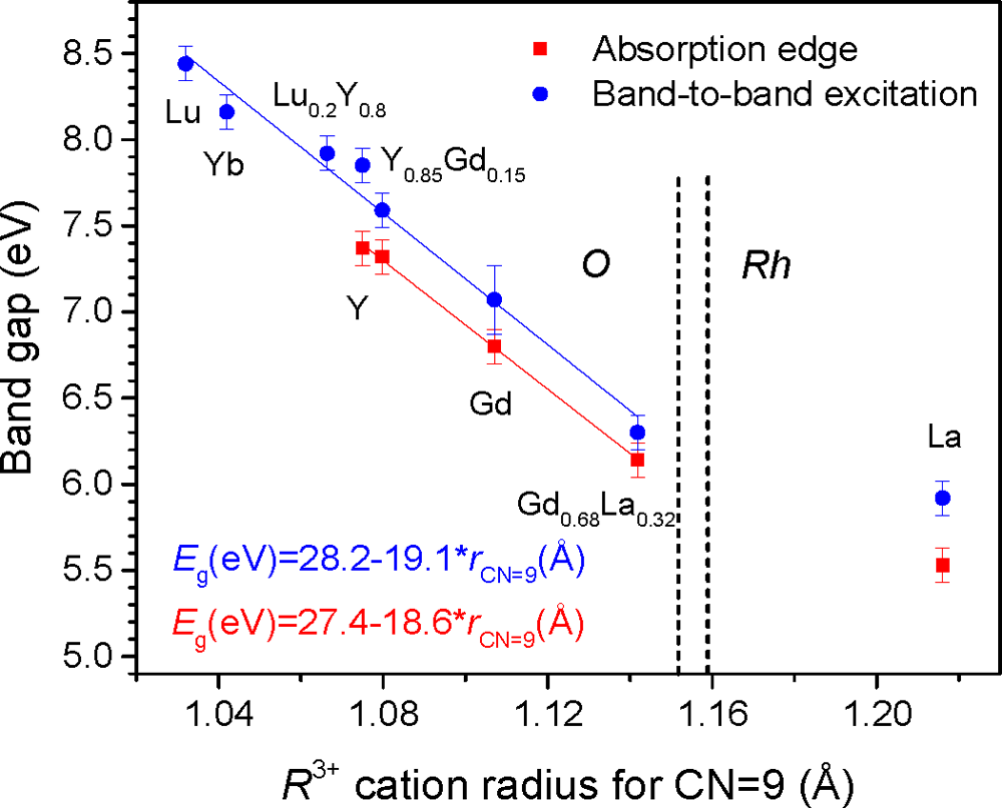
Dependences of experimentally determined band gap values of the RAlO_3_ perovskites on the ionic radius of R^3+^ cation (see Table S3 and the text for details). Solid lines represent linear fits in the composition range of the orthorhombic (*O*) structure.

The optical absorption spectra of single-crystalline RAlO_3_ are presented in Figure 2, whereas corresponding Tauc plots and extrapolations constructed with *n* = 2 and *n* = 1/2 are given in Figure S3. The band gap values estimated from the Tauc plots are shown in Figure 3 as a function of the mean ionic radius of the R cation and also given as numerical data in Table S3. The band gap values for Figure 3 were chosen from the Tauc plots with *n* = 1/2 or 2, dependent on the type of the band gap (direct or indirect) of RAlO_3_ crystals determined in calculations.

As Figure 2 shows, among the studied crystals, the nearest absorption edge at about 5.0 eV can be identified for YbAlO_3_. However, the optical absorption measurements of the YAlO_3_:Yb(4%) crystal reveal a strong band at 5.7 eV obviously caused by Yb^3+^ ions. The spectral position of this band is typical for absorption bands formed by the O^2–^ →Yb^3+^ charge transfer (CT) transitions in oxide hosts.^40−42^ Therefore, we suppose that the edge-like absorption observed for the YbAlO_3_ crystal (see corresponding curve in Figure 2) is formed by an intense absorption band caused by CT transitions from 2p states of O^2–^ to 4f states of Yb^3+^ ions. Such interpretation of the absorption edge of YbAlO_3_ is further confirmed by the luminescence excitation spectra (corresponding data will be analyzed in 3.2 Luminescence Excitation by Synchrotron Radiation).

After exclusion of the O^2–^ → Yb^3+^ CT band in YbAlO_3_, the smallest *E*_g_ value of 5.5–5.7 eV identified by absorption is that for the LaAlO_3_ crystal. It should be mentioned here that pure LaAlO_3_, at room temperature, represents the rhombohedral (*Rh*) structure type, while Gd_0.68_La_0.32_AlO_3_ and others from Table S3 are orthorhombic (*O*).^7^ In such a way, the band gap data for Gd_0.68_La_0.32_AlO_3_ are well in line with a general tendency of *E*_g_ lowering with R^3+^ cation radius in the frames of orthorhombic structure shown in Figure 3.

LuAlO_3_ undoubtedly has the largest among the studied crystals value of *E*_g_ ≥ 8.0 eV; however, its precise value was impossible to estimate from our absorption measurements because the optical density range limit of about 3.0 was already reached at 7.8 eV.

### 3.2 Luminescence Excitation by Synchrotron Radiation

**Figure 4:**
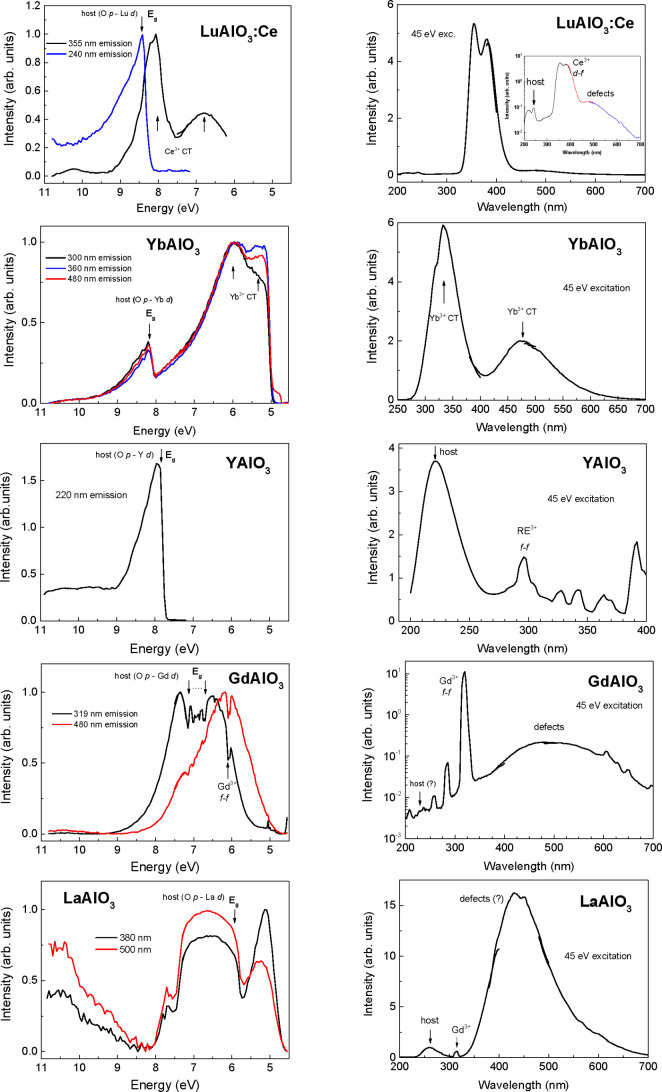
Excitation (left) and emission (right) spectra of RAlO_3_ crystals measured at 10 K. The corresponding wavelength (energies) of the emitting and exciting photons are indicated.

The band gap values of RAlO_3_ compounds can be determined from the excitation spectra of their intrinsic luminescence of recombination type. It is well-known that the spectral components of intrinsic (host-related) luminescence of oxide compounds usually reveal a steep rise followed by a relatively sharp peak in their excitation spectra.^43^ Regardless of possible contribution from the excitonic effects, such peculiarities definitely point to the energy threshold of band-to-band transitions in the excitation spectra of intrinsic recombination luminescence. The above-mentioned peculiarities of the excitation spectra can be used for an approximate (within 0.1–0.2 eV accuracy) estimation of the *E*_g_ values of oxide crystals (see Spassky *et al*.^44^ and references therein). According to such an estimation technique, the high-energy end of the steep-rise region of luminescence efficiency of the crystal indicates the *E*_g_ position. Use of this approach most probably overestimates slightly the band gap energy as compared with the absorption measurements data. The *E*_g_ values of RAlO_3_ crystals determined within this approach are indicated in Figure 4 by vertical arrows and are listed also in Table S3. Spectroscopic data of several Mn-doped (Y, Gd, La, Lu)AlO_3_ solid solution crystals (see Figure S4) reveal *E*_g_ positions which are intermediate between values for the corresponding pure crystals (see Table S3).

Beside identification of *E*_g_ positions, we would like to omit detailed analysis of the excitation and emission spectra of the crystals under synchrotron radiation. The features related to defects and intentional and unintentional dopants in the crystals are beyond the scope of the present paper and should be discussed elsewhere. We would like to note only that our results obtained for YbAlO_3_ allow us to separate unambiguously the *E*_g_ position caused by the band-to-band transitions (to 4d states of Yb^3+^) and the excitation/emission bands related with CT transitions (to/from 4f states of Yb^3+^).

Results presented in Figure 3 indicate that independent of the *E*_g_ estimation method, either from the absorption spectra or luminescence excitation spectra, the same relative changes on crystal composition are observed. These changes can be approximated well enough by a linear dependence on mean ionic radius of R^3+^ cation, at least in the composition range of the orthorhombic structure.

As follows from the data presented in Figure 3, the *E*_g_ value of RAlO_3_ perovskites gradually decreases from ∼8.5 to ∼5.5 eV with the increase of cationic radius, i.e., in the sequence of R cations Lu → Yb → Y → Gd → La. Such a wide (∼3 eV) variation obviously provides a strong potential for the BGE in RAlO_3_ perovskites.

### 3.3 Applying the BGE to Far-Red Emitting (Y,Gd,La)AlO_3_:Mn^4+^ Phosphors

#### 3.3.1 XRD Characterization

According to XRD examination, as-prepared Y_1–*x*_Gd_*x*_AlO_3_:Mn^4+^ (*x* = 0, 0.2, 0.4, 0.6, 0.8, 1.0) and Gd_1–*y*_La_*y*_AlO_3_:Mn^4+^ (*y* = 0.2, 0.3, 0.4) materials adopt orthorhombic *Pbnm* perovskite structure isotypic with GdFeO_3_. Apart of the main perovskite phase, the samples synthesized contain a minor amount of the monoclinic R_4_Al_2_O_9_ phase and traces of R_3_Al_5_O_12_ garnet phase (see Figures S5 and S6 for an example). A minor amount of the monoclinic phase in the studied samples even without the garnet phase is definitely due to the R-rich composition used for their synthesis (see 2.1 Sample Preparation and Experimental Methods for details). Exemplary graphical results of simultaneous two- and three-phase Rietveld refinement for some Y_1–*x*_Gd_*x*_AlO_3_:Mn^4+^ and Gd_1–*y*_La_*y*_AlO_3_:Mn^4+^ samples are shown in Figures S5 and S6.

Obtained structural parameters of the Y_1–*x*_Gd_*x*_AlO_3_:Mn^4+^ series synthesized (Table S4) agree well with the literature data for the nominally pure YAlO_3_ and GdAlO_3_ compounds,^7^ as well as for the mixed Y_0.5_Gd_0.5_AlO_3_ orthoaluminate,^45^ thus proving the formation of a continuous solid solution with orthorhombic perovskite structure in the YAlO_3_–GdAlO_3_ system. In contrast, in the GdAlO_3_–LaAlO_3_ system, two types of solid solutions with orthorhombic and rhombohedral perovskite structures can be formed.^7^ All three Gd_1–*y*_La_*y*_AlO_3_:Mn^4+^ materials used in the present work fall in the orthorhombic perovskite region, and their structural parameters (Table S4) are in good agreement with earlier published data for the corresponding Gd_1–*y*_La_*y*_AlO_3_ compositions.^7^

**Figure 5:**
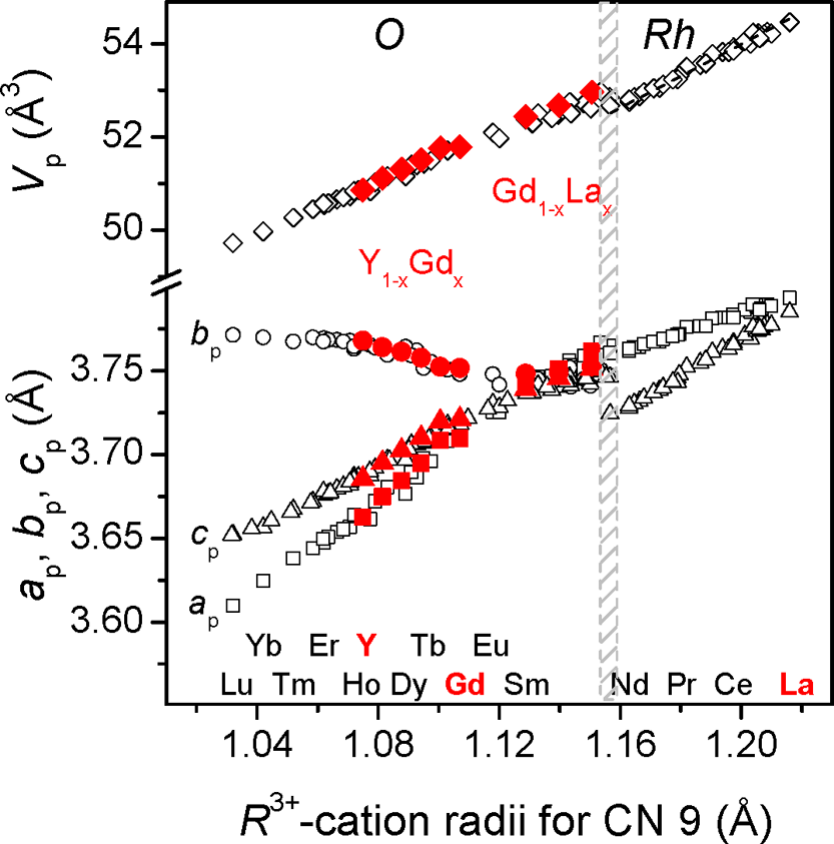
Normalized lattice parameters and unit cell volumes of rare earth aluminates versus ionic radius of R^3+^ cations (adapted from Vasylechko *et al*.^7^). Lattice parameters and unit cell volumes of orthorhombic (*O*) and rhombohedral (*Rh*) structures are normalized to perovskite (*P*) ones as follow: *a*_p_ = *a*_or_/√2, *b*_p_ = *b*_or_/√2, *c*_p_ = *c*_or_/2, *V*_p_ = *V*_or_/4; *a*_p_ = *a*_rh_/√2, c_p_ = *c*_rh_/√12, *V*_p_*= V*_rh_/6. The hatched area shows the phase-separation region. Solid red symbols represent the data for Y_1–*x*_Gd_*x*_AlO_3_:Mn^4+^ and Gd_1–*x*_La_*x*_AlO_3_:Mn^4+^ materials from the present study.

An analysis of the structural parameters shown in Table S4 indicates that unit cell volume of the Y_1–*x*_Gd_*x*_AlO_3_:Mn^4+^ and Gd_1–*y*_La_*y*_AlO_3_:Mn^4+^ perovskite structures systematically enhances with increasing of *x*(*y*) value in both series investigated. This fact is explained by increasing radii of R^3+^-cations (CN = 9): *r*(Y^3+^) = 1.075 Å, *r*(Gd^3+^) = 1.107 Å, and *r*(La^3+^) = 1.216 Å according to Shannon’s scale.^46^ The results obtained are in excellent agreement with numerous literature data for the related materials. Thus, Figure 5 represents a plot of unit cell dimensions of rare earth aluminates versus ionic radius of R^3+^ cations, in which our results for the Y_1–*x*_Gd_*x*_AlO_3_:Mn^4+^ and Gd_1–*y*_La_*y*_AlO_3_:Mn^4+^ series are compared with the reported data for RAlO_3_ compounds and diverse mixed R_1–*x*_R′_*x*_AlO_3_ aluminates.^7^

#### 3.3.2 TSL Studies above Room Temperature

When the RAlO_3_ perovskite is doped with manganese in relatively small concentration, the Mn^4+^ ions occupying the aluminum octahedra are usually observed without any codoping.^4,47−49^ As has been shown previously, Mn^4+^ in the perovskite host lattice, like YAlO_3_, can be easily photoionized already by visible blue-green light (via the Mn^4+^ → Mn^5+^ + *e*^*–*^ process) that proceeds simultaneously with the accumulation of the released electrons on the intrinsic (native) point defects and Mn^4+^ ions (Mn^4+^ + *e*^*–*^ → Mn^3+^) acting as deeper electron traps.^4,50,51^ It is considered that the intrinsic traps acting in this case have an *electron-* rather than a *hole*-related origin.^50−52^ Under subsequent thermal stimulation, the material produces an efficient far-red glow at about 715 nm caused by Mn_Al_^4+^ ions (the ^2^E → ^4^A_2_ transition).^47,48,50,51^ Exactly such phosphors emitting far-red or near-IR light have recently become of high interest as converting phosphors in solid-state lighting for indoor plant growth; night-vision surveillance; environment inspection; and, in particular, for *in vivo* biomedical applications.^53−60^

**Figure 6:**
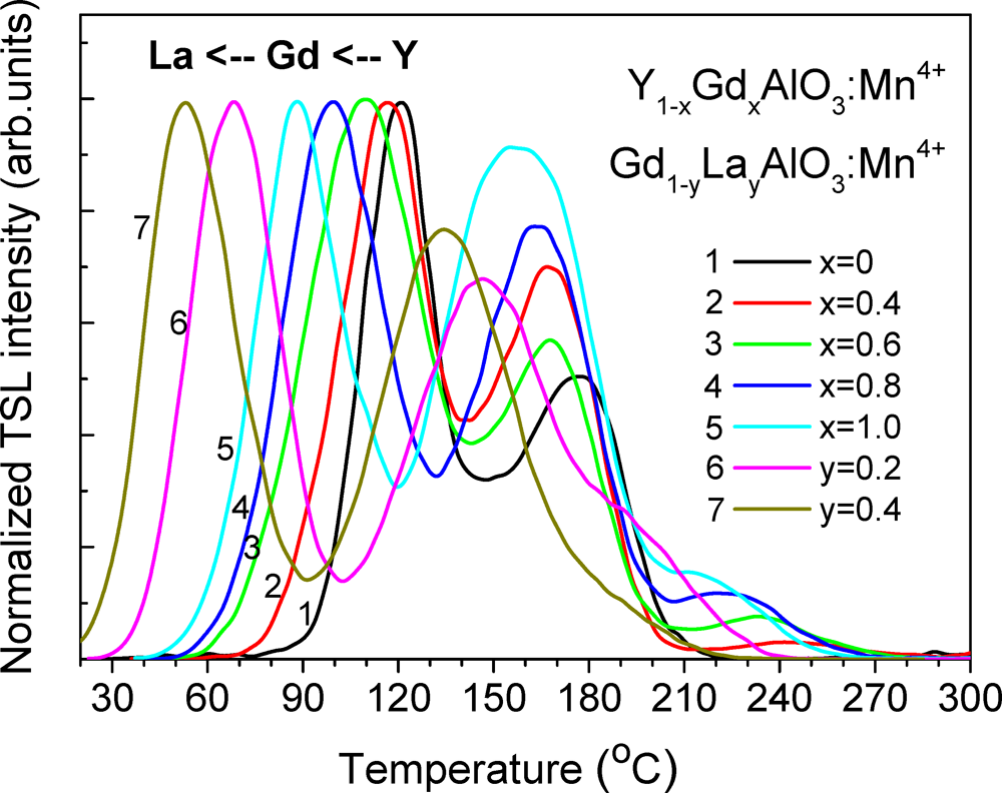
TSL curves of the (Y,Gd,La)AlO_3_:Mn^4+^ phosphors after blue-green laser illumination at room temperature with heating rate of 0.5 °C/s.

The origin of the traps responsible for the radiation-induced coloration as well as the high-temperature TSL of YAlO_3_ and related perovskite crystals is not postulated unambiguously and has been a subject of discussion for a long time (see, e.g., refs (61−66)). Supposing that all the RAlO_3_ perovskites have the same type of intrinsic electron traps, probably connected with R_Al_ antisite defects,^64−67^ one can expect that their energetic depths should be strongly dependent on the crystal composition. Our TSL results shown in Figure 6 confirm such an assumption: when the R cation is gradually replaced from Y to Gd and next to La, a *similar* structure of TSL curves is maintained; however, a systematic *shift* of the peak maxima toward lower temperature is clearly observed. Such a shift evidently correlates with the above presented decreasing of the RAlO_3_ band gap in the R sequence Y → Gd → La (taking into account the TSL results for the (Y–Lu)AlO_3_:Mn^2+^ crystals presented by Zhydachevskyy *et al*.,^68^ one can also expand this sequence of the *E*_g_ decreasing to Lu → Y → Gd → La). The data presented in Figure 6 definitely indicate a systematic lowering of the depths of the traps related to the TSL peaks in the Lu → Y → Gd → La sequence of cations.

**Figure 7:**
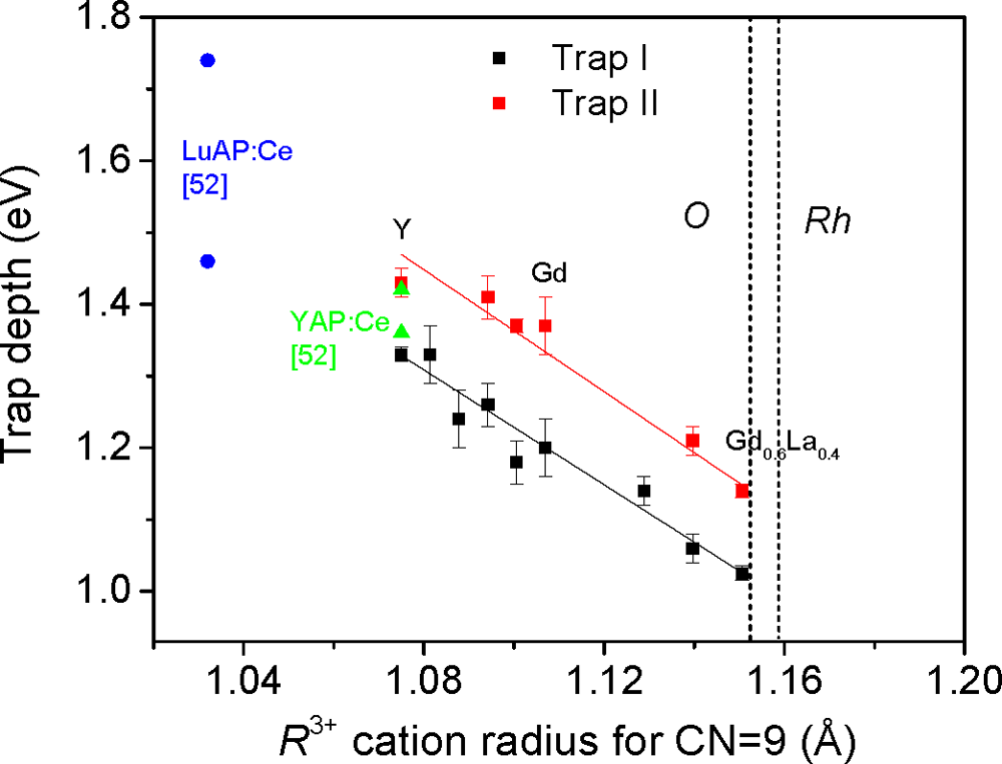
Activation energy of the intrinsic traps in the studied (Y,Gd,La)AlO_3_:Mn^4+^ phosphors versus mean ionic radius of R^3+^ cations (see text for details). Solid lines represent linear fits for the corresponding trap depths. Activation energies of main electron traps for YAlO_3_:Ce and LuAlO_3_:Ce from Wojtowicz *et al*.^52^ are also given for comparison.

To analyze such a tendency quantitatively, we have estimated the depths of acting traps from the TSL data presented in Figure 6. The trap depths were estimated by the initial rise method as shown in Figure S7. Results of the estimations are collected in Figure 7, where the data for YAlO_3_:Ce and LuAlO_3_:Ce from Wojtowicz *et al*.^52^ are also given. As is shown in Figure 7, the depth of the shallower trap (marked by us as trap I) decreases from 1.46 to 1.03 eV, and that for the deeper trap (trap II) decreases from 1.74 to 1.14 eV, when content of the R cations changes from Lu to Gd_0.6_La_0.4_.

Note that the above-mentioned results demonstrate how the BGE approach can be applied on purpose to move from the long-time storage phosphor, like YAlO_3_:Mn,^5,49,50,67^ to the efficient persistent luminescence phosphor, like (La–Gd)AlO_3_:Mn (known from Du *et al*.).^55^ At the same time, it is obvious that the following questions should be answered in order to clarify the origin of the tendency observed in TSL. First, is the observed decrease of the trap depth related only to the lowering of the crystal band gaps in the mentioned sequence of R cations? Second, does the depth (i.e., the energy distance from the defect level in the band gap to the CB minima) of the electron trap (typical for RAlO_3_ crystal) change (lower) in this sequence of R cations? These questions will be analyzed in detail in the electronic structure computational studies presented in the following section.

### 3.4 Theoretical Calculations

#### 3.4.1 Band Gap Values

**Figure 8:**
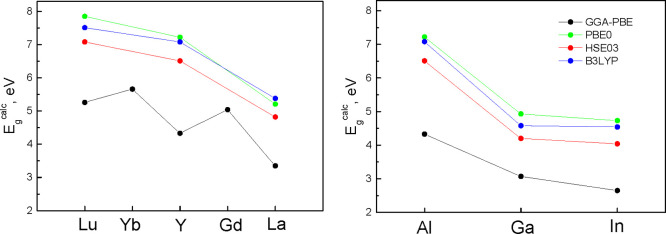
Dependencies of *E*_g_^calc^ values of RM^III^O_3_ perovskites calculated with various exchange–correlation functionals (indicated in the figure) on R (left plot, M^III^ = Al) and M^III^ (right plot, R = Y) cations.

The band gap values *E*_g_^calc^ of RM^III^O_3_ perovskites calculated with various exchange–correlation functionals are presented in Figure 8 (corresponding numerical data are given in Table S4, where also a wider set of functionals is presented in the case of YAlO_3_). These calculations were done only for the Γ point of the Brillouin zone. As Figure 8 shows, regardless of what exchange–correlation functional is used, the calculations, in general, give the same tendencies in the band gap shift dependent on R = La, Y, and Lu cations, which were observed experimentally. As follows from Figure 3, experiments give approximately −1.7 eV band gap shift for Lu relative to Y and +1 eV for La relatively to Y, whereas −1.4 and +0.6 eV shifts are obtained in calculations for La and Lu, respectively (see Table S1).

The right part of Figure 8 demonstrates a gradual low-energy shift of the band gap energies for the sequence of M^III^ cations Al → Ga → In. Such a tendency is well in line with the results of previous computational studies for garnets.^12,16^ Therefore, our calculations confirm also perspectives of the BGE approach in RM^III^O_3_ perovskites via substitution of M^III^ cations.

Figure 8 also clearly indicates that for such cations, the use of various functionals can only increase the *E*_g_^calc^ values (by the same value for all R and M^III^); however, the cationic-dependent tendencies are kept practically the same. This fact implies that if only the differences in *E*_g_^calc^ are the main focus of analysis (for example, in the BGE problem), the use of GGA-PBE functional will have the same accuracy as that of other functionals; however, a valuable gain in the computational efficiency is provided. In our study, the calculations of the defect levels in the crystal band gaps were done with use of the GGA-PBE functional because of its computational efficiency.

The data in Figure 8 indicate that *E*_g_^calc^ values for R = Gd and Yb are not in line with experimental tendencies. It is obvious that *E*_g_^calc^ values for these two cations are not in line, most probably because of the use of the LSDA+U approximation. These *E*_g_ values were obtained with Hubbard U parameters (see Table S1) which provide the absence of Gd f and Yb f states at the band edges of corresponding crystals. Further adjustment of the band gaps for GdAlO_3_ and YbAlO_3_ to experimental values requires additional calculations. For this reason, we model the BGE effect in computational studies considering only perovskites with R = Y and La. However, it would not be hard to extend our approach and inferences to perovskites with other R cations.

It is well-known that fitting of the calculated *E*_g_ to experimental values by means of searching through appropriate exchange–correlation functionals is a procedure well-justified only for the band-periodic electron states of crystals or solid solutions, and such procedure is barely applicable for the states of defects in the crystal band gap. Therefore, in our computational studies presented in the next subsection, we analyze the results for the defect level positions in the crystal band gaps obtained with the GGA-PBE exchange–correlation functional and without further fitting of *E*_g_^calc^ to experimental values. Such an approach has been generally used in computational studies of defects in RAlO_3_,^14,69−77^ and its adequacy has never been in question.

#### 3.4.2 Intrinsic Point Defects and Their Trap Depths

Despite the main experimental tendencies of the crystal band gaps and trap depths obtained for the Mn^4+^-doped samples (see previous sections), we do not consider in calculations presented here any Mn-related defects in perovskites. We believe that the TSL properties (namely, traps I and II) analyzed in the previous section are formed by native (intrinsic) defects of the crystal hosts and are not related to Mn impurity ions (for more details, see Przybylińska *et al*.^78^ and references therein). Owing to the experimental results reported in refs (64−67), these are *electron* traps related, most probably, to Y_Al_ antisites stabilized by some other intrinsic point defects. For this reason, along with considering in calculations the most commonly encountered single native and substitution defects, we focus our attention on Y_Al_-containing complex defects in YAlO_3_.

**Figure 9:**
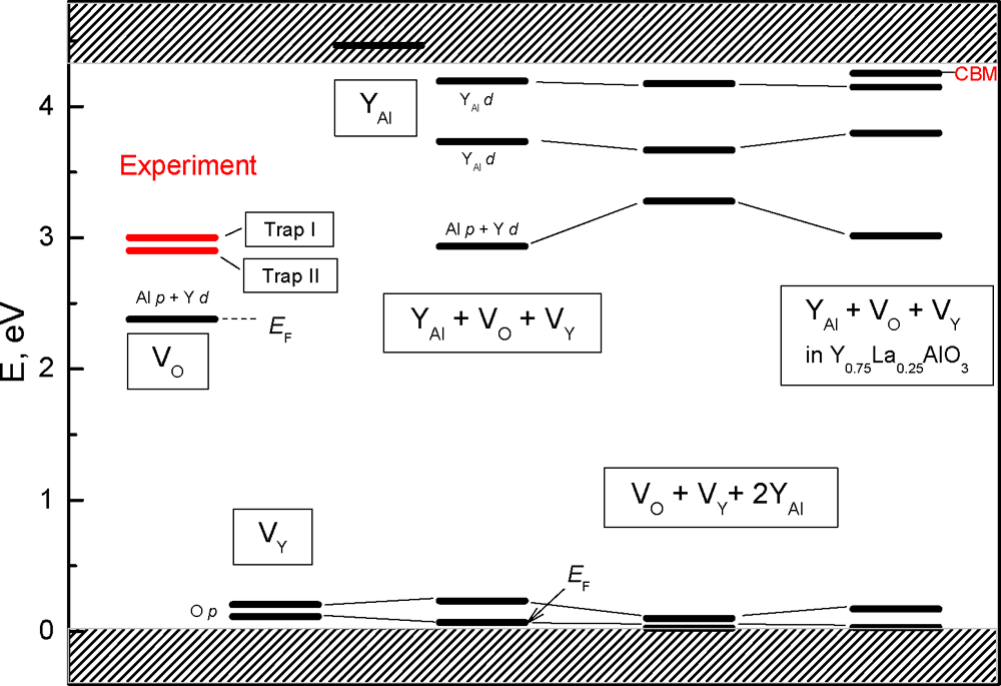
Experimental trap depths (red bars) and calculated energy levels (black bars) of some defects (see text for details) in the band gap of YAlO_3_ crystals. Atomic orbital characters for the defect levels and calculated Fermi energies (*E*_F_) are indicated as well; *E*_F_ = 0 if not indicated.

The calculated trap depths of all studied defects in YAlO_3_ are presented in Table S5. The depths were obtained as the energy difference between the defect level position and the VB maximum, *E*_D_ – *E*_v_ for hole traps, and correspondingly, as *E*_c_ – *E*_D_ for the electron traps (assuming that if the defect level *E*_D_ lies closer to the CB minimum, it can act as an electron trap, and if the level is closer to VB maximum, this defect can form a hole trap). As our calculations show (see Table S5), the calculated trap depths of some defects in YAlO_3_ substantially depend on type of spin polarization (different spin polarizations are denoted in Table S5 as α and β). However, for defects which are the focus of analysis of this paper (presented in Figure 9), such difference is negligible (less than 10^–3^ eV). For this reason, in order to avoid additional complication of analysis, herein we consider trap depths calculated only for one (α) spin polarization.

It should be noted that the determination of energy position of defect levels in the crystal band gap by the DFT method as well as the use of finite-size super cells may lead to some inaccuracy in the obtained trap depths. However, the super cell size, computational parameters, and approximations of the method applied here are typical for the present-day computational modeling of the electronic structure of YAlO_3_ crystals with defects (see, e.g., ref (77)). Taking into account the possible inaccuracy of the obtained calculation results, we can make only *assumptions* regarding the role of specific defects in the formation of the TSL properties of RAlO_3_ crystals. The assumptions can be formulated as the following.

As follows from the analysis of the TSL data presented above or available literature data,^50,52,78,79^ the defects, which form the main high-temperature TSL peaks in YAlO_3_ and YAlO_3_:Mn^4+^, usually have depths from 1.20 to 1.43 eV. It is commonly assumed that these defects are electron traps.^50,64−67,78^ According to Table S5, none of the single point defects like Y_Al_, V_O_, O_*i*_, or V_Y_ have energy levels of appropriate depth with respect to the CB to be responsible for the high-temperature TSL or the radiation-induced coloration of YAlO_3_ crystals stable at room temperature. However, as our calculation results show, the trap depths of single defects can be considerably changed if the defects are complexed with each other. For example, the deep energy level of V_O_ (electron trap of 2.38 eV depth) becomes much shallower (0.93 or 0.47 eV) when the V_O_ is complexed with cation vacancy (V_Al_ or V_Y_, respectively). At the same time, the O_*i*_ interstitial, which is a shallow hole trap when existing alone, can serve as an electron trap of 0.65 eV depth when complexed with Y_Al_ antisite (see Table S5 and Figure S8).

As it is known from experiments, the high-temperature TSL and the radiation-induced coloration of YAlO_3_ is related to the yttrium over aluminum excess (Y/Al > 1).^51,61−64^ At the same time, the high-temperature oxidizing annealing (related to oxygen excess in the crystal samples) produces strong coloration of the crystal.^61−63,80^ Therefore, our calculations as well as existing experimental data indicate that point defects related to yttrium excess, like Y_Al_, and related to oxygen excess, like O_*i*_ and/or V_Y_(V_Al_), are the core components of complex defects that can form the TSL properties of YAlO_3_ crystals above room temperature.

As our calculations show, among all the defect complexes considered in the present work, only the (Y_Al_ + V_O_ + V_Y_) complexes have an electron trap depth (1.41 eV) close to the experimental values for trap II (see 3.3.2 TSL Studies above Room Temperature). For this reason we consider the levels of such complex defects in more detail in Figure 9 (corresponding schemes for other defects are given in Figure S8).

As Figure 9 shows, the (Y_Al_ + V_O_ + V_Y_) defect has several levels in the crystal band gap. Two of them are related to V_Y_ vacancies and can form shallow traps for the holes. It is clearly seen that the level of the (Y_Al_ + V_O_ + V_Y_) defect of 1.41 eV depth originates from the oxygen vacancy V_O_. However, a single V_O_ forms a much deeper electron trap of 2.38 eV (a similar result is typically obtained in the DFT-based calculations).^73,74^ In such a way, one can assume that the electron trap of 1.43 eV acting in TSL of YAlO_3_ (trap II) can be attributed to the V_O_-related level of the (Y_Al_ + V_O_ + V_Y_) complex defect in yttrium aluminum perovskite. However, we must be aware that it is just a tentative assignment based on the presented results because not all possible combinations of the single defects have been considered.

Two more shallow electron traps (with 0.606 and 0.146 eV depths) of the (Y_Al_ + V_O_ + V_Y_) defect are related to the electronic states of Y_Al_ antisite substitution (as Figure 9 shows, a single Y_Al_ has no levels in the crystal band gap). These electron traps are too shallow to correspond to the (Y_Al_ + V_O_) complexes observed previously by the EPR technique by Laguta *et al*.^65^ (owing to the thermal stability of the centers reported by Laguta *et al*.,^65^ their depth should be about 0.9–1.1 eV). However, the Y_Al_-related levels of the (2Y_Al_ + V_O_) complex of the 0.91 eV depth for electrons (see Table S5) can exactly be the Y_Al_-related electron traps known from Laguta *et al*.^65^

The electron trap I of 1.33 eV depth found in experiments for YAlO_3_ can be presumably formed by a complex like (Y_Al_ + V_O_ + V_Y_) with an up-shifted V_O_-related level (experimental upshift from trap II level to trap I is 0.1 eV). Such a shift can arise, for example, in the same complex, but with slightly different spatial configuration of Y_Al_, V_O_, and V_Y_ constituents. An upshift of V_O_-related level (by 0.39 eV, see Figure 9) was obtained in calculations for the (V_O_ + V_Y_ + 2Y_Al_) complex, in which two Y_Al_ substitutions are on the Al positions closest to V_O_ and V_Y_. However, the trap depth of this level (see Table S5) is 1.015 eV and does not fit well the experimentally determined depth of trap I. At the same time our calculations of the (V_O_ + V_Y_ + 2Y_Al_) complex reveal also some down-shift of the Y_Al_-related level (see Figure 9), so it can tentatively also correspond to some type of Y_Al_ trapping centers observed by the EPR technique by Laguta *et al*.^65^

Our calculations also indicate that doping of YAlO_3_ with La can make the electron traps shallower. As Figure 9 shows, the V_O_-related level of the (Y_Al_ + V_O_ + V_Y_) complex defect (associated with the electron trap II, see above) in Y_0.75_La_0.25_AlO_3_ has the depth of 1.24 eV, which is 0.17 eV smaller than the depth of the corresponding level in YAlO_3_ (1.41 eV). Such a 0.2 eV shift of trap depth is consistent with the experimental results on the cation-related TSL activation energy shifts. For example, trap II in Gd_0.8_La_0.2_AlO_3_ is by 0.2 eV shallower than that in YAlO_3_ (see Figure 7).

According to Figure 9, the 0.17 eV decrease in trap depth of the V_O_-related level of the (Y_Al_ + V_O_ + V_Y_) defect in Y_0.75_La_0.25_AlO_3_ is related to the decrease (by 0.07 eV) of the level position with respect to the CB minimum of the mixed crystal, as well as to the lowering (by 0.1 eV) of the CB minimum (*E*_c_) of Y_0.75_La_0.25_AlO_3_ relative to YAlO_3_ case. The mechanism of such *E*_c_ lowering may be explained as follows. A single La_Y_ defect in YAlO_3_ creates defect levels of La d character just below the CB of the crystal (see Figure S8). If the concentration of La_Y_ defects is low, their mutual influence is negligible and they can form only defect levels in the crystal band gap. If concentration of La_Y_ defects is sufficiently high, as for example in Y_0.75_La_0.25_AlO_3_ solid solution, the defect levels can already form periodic bands in the reciprocal space lying below *E*_c_ of YAlO_3_ crystal. In real space, the electronic states which correspond to these bands may form quasi-infinite regions for spatial movement of free electrons. For this reason, the appearance of La_Y_-related bands below *E*_c_ of YAlO_3_ may be regarded as lowering of the CB minimum of the crystal.

Therefore, it could be argued that our calculations provide direct computational evidence for the possibility of BGE in RAlO_3_ perovskites using R = Y and La as an example. Extending the analysis to RM^III^O_3_ perovskites with other R and M^III^ cations, as well as consideration of a wider set of defects, should be a subject of further computational studies.

## 4 Conclusions

The following observations and conclusions result from the complex experimental and theoretical studies presented above:

(1) Depending on the R and M^III^ cations, the RM^III^O_3_ perovskite crystals may be direct- (LuAlO_3_, GdAlO_3_, LaAlO_3_, YbAlO_3_, and YGaO_3_) or indirect-band gap materials (YAlO_3_, LaAlO_3_, and YInO_3_).

(2) The gradual decrease of band gap value (*E*_g_) of RAlO_3_ perovskites from ∼8.5 to ∼5.5 eV with increase of cationic radius, i.e., in sequence of R cations Lu → Yb → Y → Gd → La, has been shown experimentally using both the optical absorption measurements in VUV spectral range and the spectra of luminescence excitation by synchrotron radiation. Such a wide (∼3 eV) variation of band gap values obviously provides a strong potential for the band gap engineering of RAlO_3_ perovskite compounds.

(3) The DFT electronic structure calculations confirm perspectives of the BGE approach in perovskites: the band gaps of RM^III^O_3_ crystals gradually decrease in the Lu–Y–La sequence of R cations and Al–Ga–In sequence of M^III^ cations.

(4) When the R cation of RAlO_3_ is gradually replaced from Y to Gd and next to La, a *similar* structure of thermally stimulated luminescence curves (two main peaks associated with the traps denoted as I and II) is systematically shifted toward lower temperature. Such a shift indicates a lowering of the energy depth of acting traps in the Y → Gd → La sequence of cations. When the content of the R cations changes from Lu to Gd_0.6_La_0.4_, the depth of the shallower trap I decreases from 1.46 to 1.03 eV and the depth of the deeper trap decreases from 1.74 to 1.14 eV.

(5) Calculations indicate that trap depths of single-point defects Y_Al_, V_O_, O_*i*_, or V_Y_ in YAlO_3_ can be considerably changed when these defects are complexed with each other. In particular, the energy level of the Y_Al_ antisite (which has no energy levels in the band gap when alone) can be as deep as 0.9 eV with respect to the CB when Y_Al_ is complexed with a neighboring V_O_ and even of 1.0 eV when two Y_Al_ antisites are complexed with neighboring V_O_ and V_Y_, which is consistent with the experimental results reported by Laguta *et al*.^65^ The deep energy level of V_O_ (2.38 eV for electrons) became much shallower (0.93 or 0.47 eV) when the oxygen vacancy was complexed with cation vacancy (V_Al_ or V_Y_ respectively). At the same time, the energy level of the O_*i*_ interstitial (which does not have deep levels in the band gap when alone) can serve as an electron trap of 0.65 eV depth when complexed with the Y_Al_ antisite.

(6) The performed calculations allow the tentative assumption that the experimentally observed trap II (1.43 eV depth in YAlO_3_) can be formed by the V_O_-related energy level of the (Y_Al_ + V_O_ + V_Y_) complex defect which captures an electron. The shallower electron trap I (1.33 eV in YAlO_3_) presumably can be formed also by the energy level of oxygen vacancy in the same complex defect, but with somewhat different arrangement of neighboring Y_Al_ and V_Y_.

(7) Calculations provide direct computational evidence for the possibility of BGE in RAlO_3_ perovskites using R = Y and La as an example. In particular, the calculations predict shallowing of the V_O_-related level of the (Y_Al_ + V_O_ + V_Y_) complex defect in La-containing YAlO_3_ crystal by 0.17 eV, which is consistent with the experimental results regarding the cation-related shifts of TSL activation energies. The 0.17 eV decrease of the trap depth is related to the decrease (by 0.07 eV) of the level position with respect to the CB minimum and to the lowering (by 0.1 eV) of the band gap (*E*_g_) of Y_0.75_La_0.25_AlO_3_ relative to YAlO_3_.

## Supporting Information Available

Additional data including calculated electronic band structures, partial densities of states, and band gap values of RM^III^O_3_ perovskites (R = Y, La, Lu, Gd, Yb; M^III^ = Al, Ga, In); calculated energy levels and trap depths of several defects within the band gap of YAlO_3_; Tauc plots from the room-temperature optical absorption spectra of the RAlO_3_ single crystals; excitation and emission spectra of Mn-doped (Y, Gd, La, Lu)AlO_3_ solid solutions obtained under excitation by synchrotron radiation at 10 K; band gap values of RAlO_3_ crystals estimated experimentally from the VUV absorption and excitation spectra by synchrotron radiation; trap depth estimation procedure from TSL; crystal structure parameters of the Y_1–*x*_Gd_*x*_AlO_3_:Mn^4+^ (*x* = 0, 0.2, 0.4, 0.6, 0.8, 1) and Gd_1–*y*_La_*y*_AlO_3_:Mn^4+^(*y* = 0.2, 0.3, 0.4) phosphors (PDF)
Structural (cif) files of geometry optimized for perfect and defect super cells of YAlO_3_ crystal (zip)
