# Supplementary material for: Band Gap Engineering and Trap Depths of Intrinsic Point Defects in RAlO3 (R = Y, La, Gd, Yb, Lu) Perovskites
Source: J Phys Chem C Nanomater Interfaces. 2021 Nov 23;125(48):26698–710. doi: 10.1021/acs.jpcc.1c06573 (PMC8672454; doi:10.1021/acs.jpcc.1c06573)
Supplement: Supplementary file 1 — jp1c06573_si_001.pdf [file jp1c06573_si_001.pdf]

Supporting Information

Band Gap Engineering and Trap Depths of Intrinsic Point Defects in *R*AlO_3 (*R* = Y, La, Gd, Yb, Lu) Perovskites

Yaroslav Zhydachevskyy[a,b*], Yuriy Hizhnyi[c*], Sergii G. Nedilko[c], Irina Kudryavtseva[d], Vladimir Pankratov[e], Vasyl Stasiv[a], Leonid Vasylechko[b], Dmytro Sugak[b], Aleksandr Lushchik[d], Marek Berkowski[a], Andrzej Suchocki[a], and Nickolai Klyui[f,g]

[a] Institute of Physics, Polish Academy of Sciences, aleja Lotników 32/46, Warsaw 02-668, Poland

[b] Lviv Polytechnic National University, S. Bandera Str. 12, Lviv 79013, Ukraine

[c] Taras Shevchenko National University of Kyiv, Volodymyrska Str. 60, Kyiv 01033, Ukraine

[d] Institute of Physics, University of Tartu, W. Ostwald Str. 1, Tartu 50411, Estonia

[e] Institute of Solid State Physics, University of Latvia, Kengaraga Str. 8, Riga 1063, Latvia

[f] College of Physics, Jilin University, 2699 Qianjin Str., Changchun 130012, China

[g] V.E. Lashkaryov Institute of Semiconductor Physics, National Academy of Sciences of Ukraine, 41 prospekt Nauki, Kyiv 03028, Ukraine

*Email: zhydach@ifpan.edu.pl

*Email: hizhnyi@univ.kiev.ua

Table of Contents

Part 1. Calculated electronic band structures, partial densities of states and band gap values of RM^{III}O$_3$ perovskites (R = Y, La, Lu, Gd, Yb; M^{III} = Al, Ga, In).

Figure S1. Electronic band structures of perfect RM^{III}O$_3$ perovskite crystals.

Figure S2. Partial densities of states of RM^{III}O$_3$ perovskites.

Table S1. Band gap values of $RM^{III}O_3$ perovskites.

Table S2. Band gap parameters of $RM^{III}O_3$ perovskites.

Part 2. Experimental estimation of the band gap values of RAlO$_3$ crystals.

Figure S3. Tauc plots from the room-temperature optical absorption spectra of the RAlO$_3$ single crystals.

Figure S4. Excitation and emission spectra of Mn-doped (Y, Gd, La, Lu)AlO$_3$ solid solutions obtained under excitation by synchrotron radiation.

Table S3. The band gap values of RAlO$_3$ crystals estimated from the VUV absorption spectra and the excitation spectra of the host-related emission measured at using synchrotron radiation.

Part 3. Crystal structure parameters of the studied $Y_{1-x}Gd_xAlO_3:Mn^{4+}$ (x=0, 0.2, 0.4, 0.6, 0.8, 1) and $Gd_{1-y}La_yAlO_3:Mn^{4+}$ (y=0.2, 0.3, 0.4) phosphors.

Figure S5. Graphical results of Rietveld refinement for the $Y_{0.6}Gd_{0.4}AlO_3:Mn^{4+}$ sample.

Figure S6. Graphical results of Rietveld refinement for the $Gd_{0.7}La_{0.3}AlO_3:Mn^{4+}$ sample.

Table S4. Refined lattice parameters (a, b, c) and unit cell volume (V) of $Y_{1-x}Gd_xAlO_3:Mn^{4+}$ (x=0, 0.2, 0.4, 0.6, 0.8, 1) and $Gd_{1-y}La_yAlO_3:Mn^{4+}$ (y=0.2, 0.3, 0.4) perovskites.

Part 4. Estimation of trap depths from TSL.

Figure S7. Partial results demonstrating the trap depths estimated by the initial rise method in the partial cleaning procedure for the $Y_{0.2}Gd_{0.8}AlO_3:Mn^{4+}$ sample.

Part 5. Calculated energy levels and trap depths of several defects within the band gap of YAlO$_3$.

Figure S8. Calculated energy levels of several defects within the band gap of YAlO$_3$.

Table S5. Calculated trap depths of native, interstitial, substitution and antisite defects in YAlO$_3$.

Part 1. Calculated electronic band structures, partial densities of states and band gap values of $RM^{III}O_3$ perovskites (R = Y, La, Lu, Gd, Yb; M^{III} = Al, Ga, In)

$YAlO_3$ (α and β)

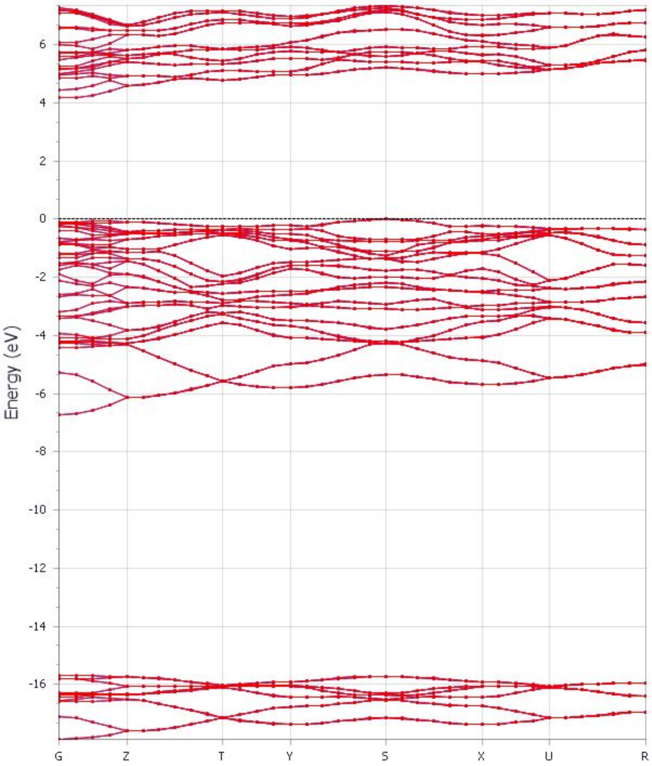

$LaAlO_3$ (α and β)

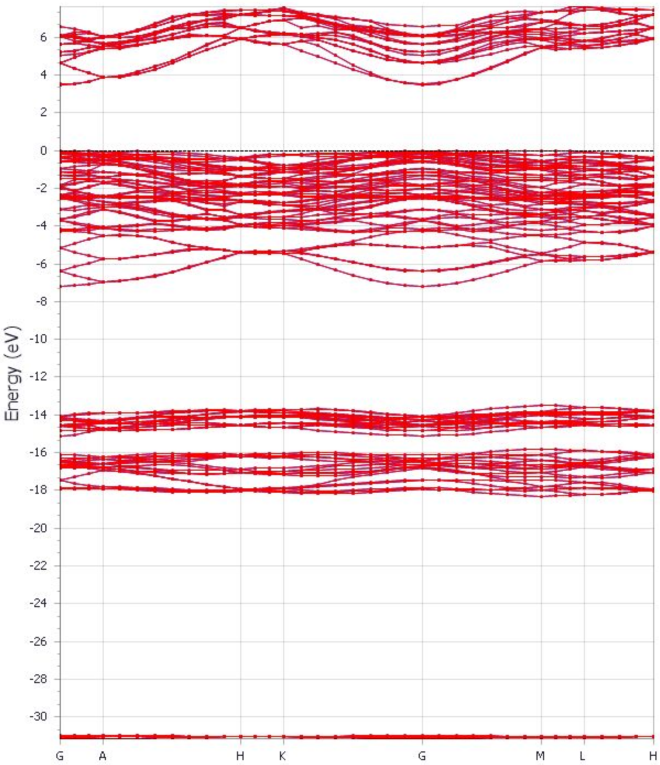

$LuAlO_3$ (α and β)

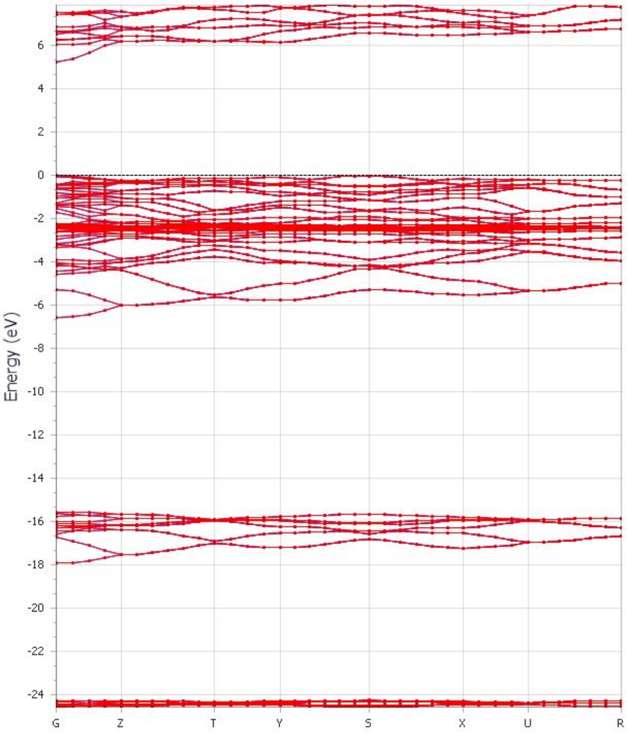

$YbAlO_3$ (- α, - β)

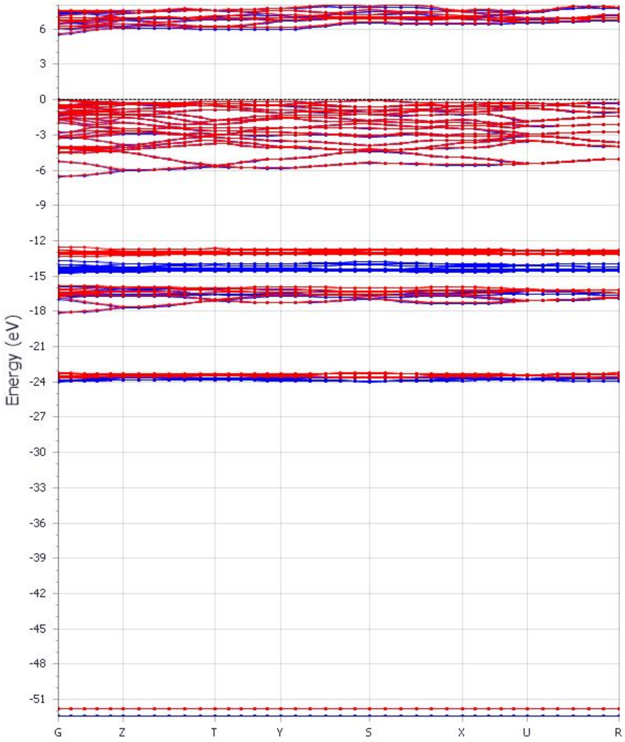

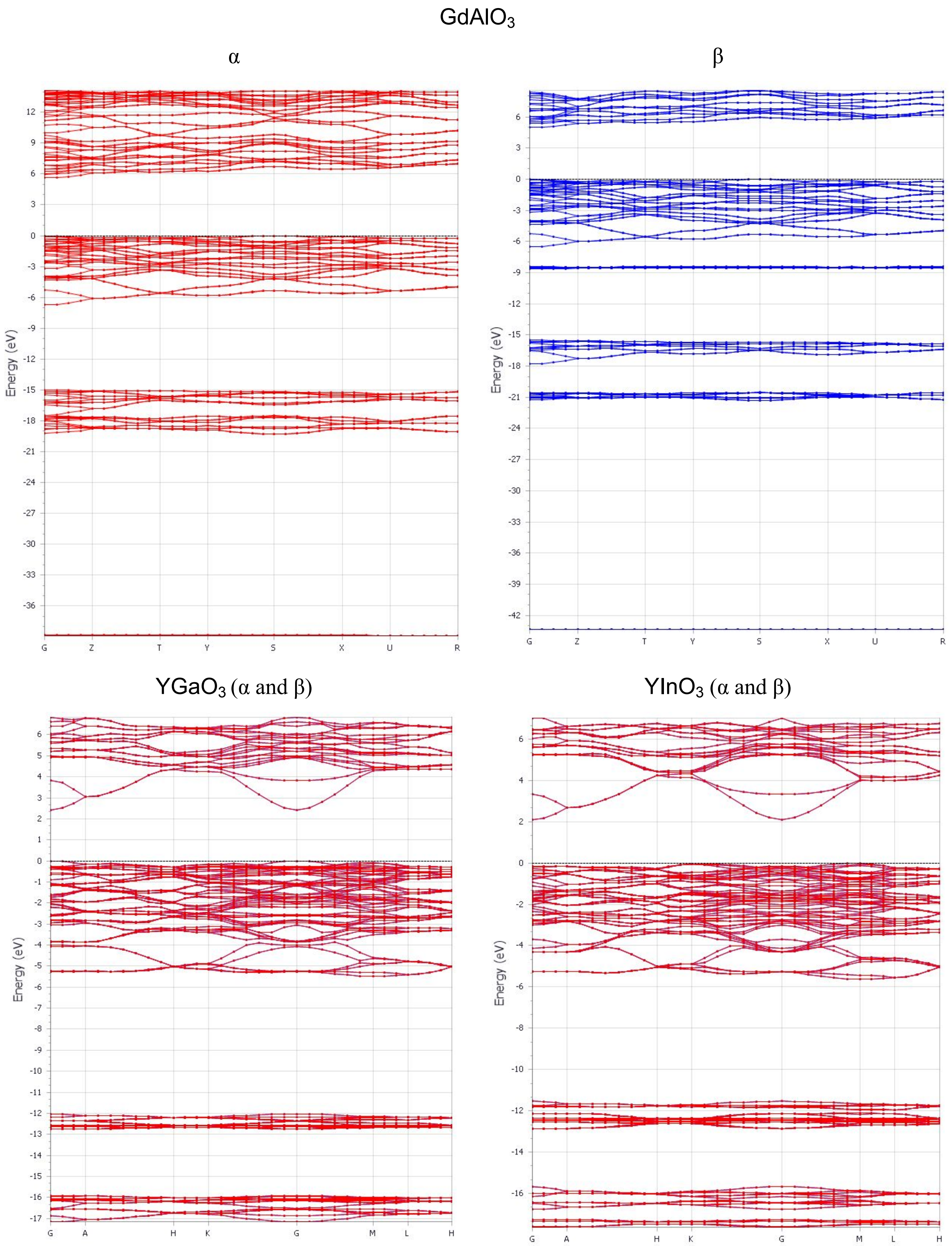

Figure S1. Energy dispersion curves $E(\mathbf{k})$ (electronic band structures) of perfect $RM^{III}O_3$ perovskite crystals calculated in reciprocal space sections between special **k**-points of the Brillouin zones (α and β denote different spin directions).

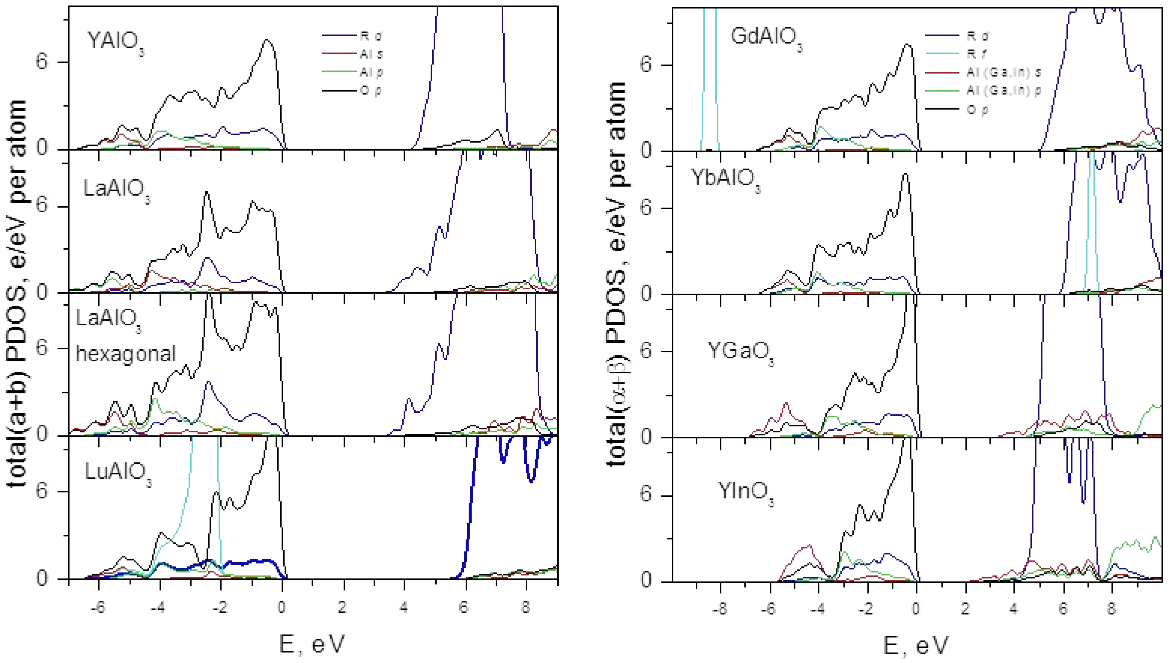

Figure S2. Calculated partial densities of states of $RM^{III}O_3$ perovskites (R = Y, La, Lu, Gd, Yb; M^{III} = Al, Ga, In).

Table S1. Calculated band gap values of $RM^{III}O_3$ (R = Y, La, Lu, Gd, Yb; M^{III} = Al, Ga, In) perovskites.

Compound	Structure type, symmetry group, source	V_{xc}	E_g, eV	Notes
$YAlO_3$	orthorhombic, Pbnm,[33]	GGA-PBE	4.86	
		GGA-RPBE	4.89	
		GGA-PW91	4.87	
		GGA-WC	4.84	
		GGA-PBESOL	4.85	
		HF-LDA	15.07	
		sX-LDA	6.85	
		PBE0	7.22	
		B3LYP	7.08	
		HSE03	6.51	
		HSE06	6.43	
$LaAlO_3$	trigonal, R-3C,[34]	GGA-PBE	3.42	
		PBE0	5.21	
		HSE03	4.82	
		B3LYP	5.38	
$LuAlO_3$	orthorhombic, Pbnm,[33]	GGA-PBE	5.49	
		PBE0	7.85	
		HSE03	7.08	
		B3LYP	7.51	
$GdAlO_3$	orthorhombic, Pbnm,[35]	GGA-PBE	5.08	Hubbard U = 6.0 eV for Gd *f*
$YbAlO_3$	orthorhombic, Pbnm,[36]	GGA-PBE	5.66	Hubbard U = 20.0 eV for Yb *f*
$YGaO_3$	hexagonal, P63CM,[37]	GGA-PBE	2.77	
		PBE0	4.93	
		HSE03	4.20	
		B3LYP	4.58	
$YInO_3$	hexagonal, P63C,[38]	GGA-PBE	2.65	
		PBE0	4.73	
		HSE03	4.04	
		B3LYP	4.54	

Table S2. Calculated band gap parameters of $RM^{III}O_3$ perovskite crystals.

Crystal	VBM (**k**-point)	CBM (**k**-point)	E_g^{direct}, eV (**k**-point)	$E_g^{indirect}$, eV (**k**-points)	ΔE_g, eV	Type of band gap
$YAlO_3$	S	Γ	4.278 (Γ)	4.173 (S → Γ)	0.105	indirect
$LaAlO_3$	M	Γ	3.521 (Γ)	3.496 (M → Γ)	0.025	indirect
$LuAlO_3$	Γ	Γ	5.261 (Γ)	---	---	direct
$GdAlO_3$	Γ	Γ	5.032 (Γ)	---	---	direct
$YbAlO_3$	Γ	Γ	5.620 (Γ)	---	---	direct
$YGaO_3$	Γ	Γ	2.435 (Γ)	---	---	direct
$YInO_3$	M	Γ	2.239 (Γ)	2.094 (M → Γ)	0.145	indirect

VBM (**k**-point in the first Brilloun zone of the crystal) - position of the VB maximum in reciprocal space;
CBM - position of the CB maximum in reciprocal space;
E_g^{direct} (**k**-point of transition) – band gap for direct transitions;
$E_g^{indirect}$ (**k**-points of initial and final states of transition) – band gap for indirect transitions;
$\Delta E_g = (E_g^{inirect} - E_g^{direct})$ – energy difference between the band gaps for direct and indirect transitions.

Part 2. Experimental estimation of the band gap values of $RAlO_3$ crystals

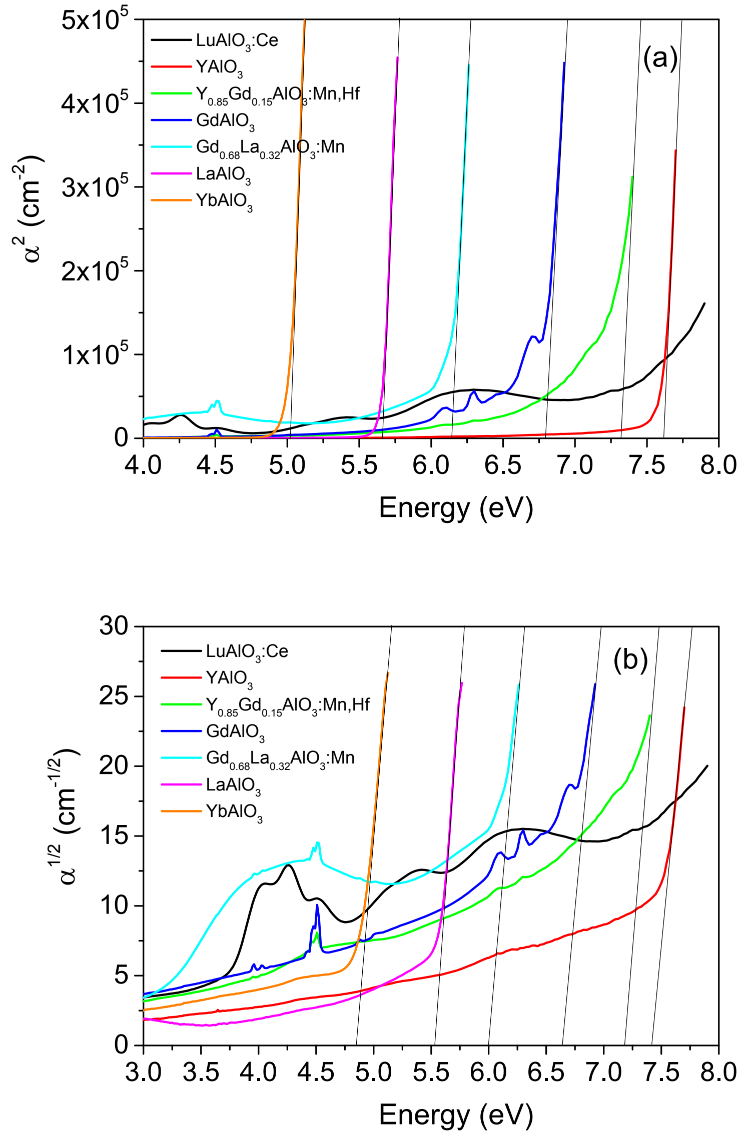

Figure S3. Tauc plots made from the room-temperature optical absorption spectra of the single crystalline $RAlO_3$ perovskites under assumption of the direct allowed (a) or indirect allowed (b) band-to-band transitions.

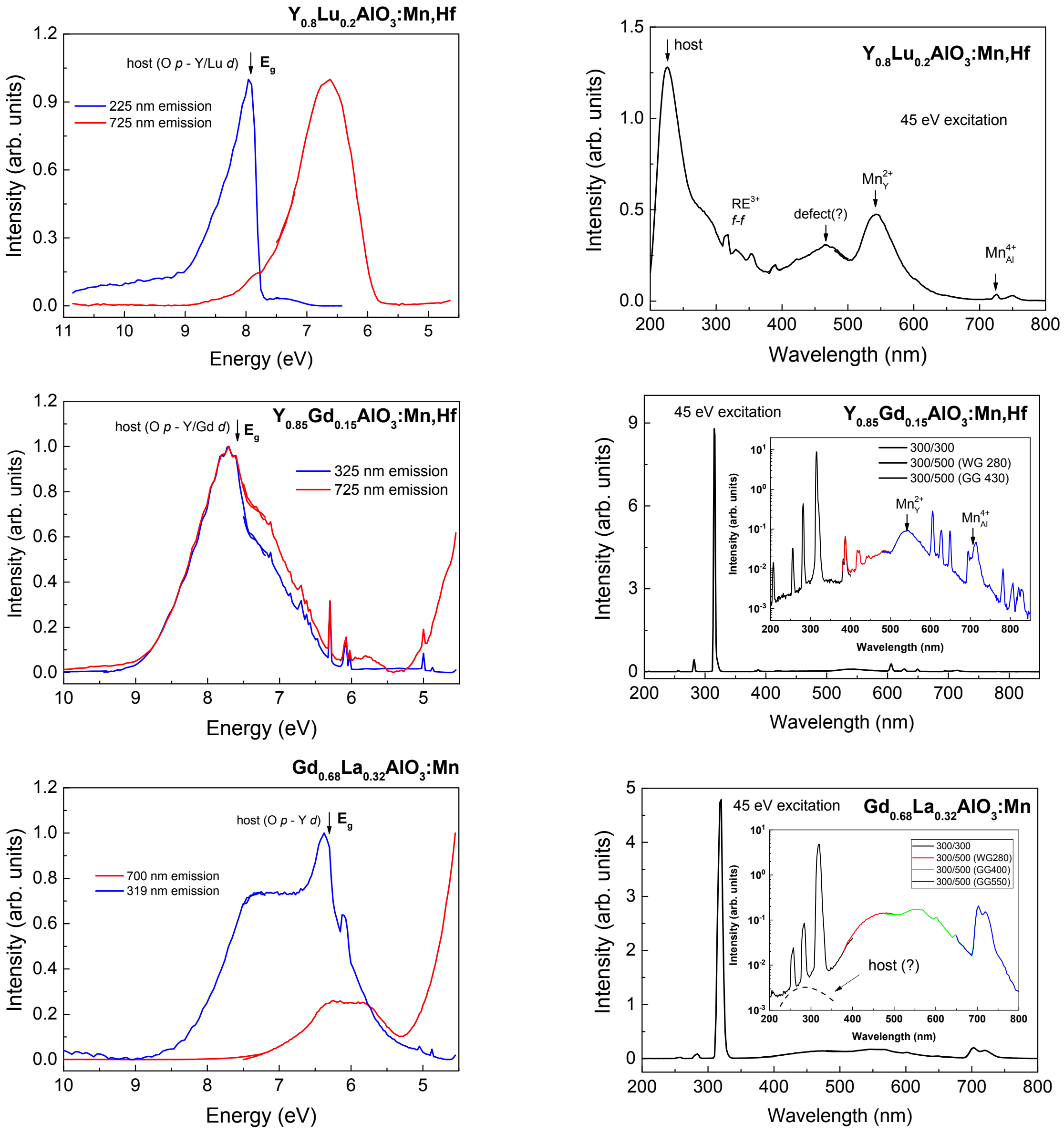

Figure S4. Excitation (left) and emission (right) spectra of Mn-doped (Y, Gd, La, Lu)AlO_3 solid solutions obtained under excitation by synchrotron radiation at 10 K, corresponding wavelength (energies) of the emitting and exciting photons are indicated.

Table S3. The band gap values E_g (eV) of $RAlO_3$ crystals estimated from the VUV absorption spectra (as shown on Figure S3) and the excitation spectra of the host-related emission measured at 10 K using synchrotron radiation (Figs. 4 and S4).

Compound	VUV absorption		Band-to-band transition excitation by synchrotron radiation
	Direct allowed transitions (n = 1/2)	Indirect allowed transitions (n = 2)	
$LuAlO_3$	≥8.0	≥8.0	8.44
$YbAlO_3$	5.02*	4.85*	8.16
$Y_{0.8}Lu_{0.2}AlO_3$			7.92
$YAlO_3$	7.60	7.37	7.85
$Y_{0.85}Gd_{0.15}AlO_3$	7.32	7.2	7.59
$GdAlO_3$	6.80	6.65	7.07
$Gd_{0.68}La_{0.32}AlO_3$	6.14	6.0	6.30
$LaAlO_3$	5.66	5.53	5.92

* the edge-like absorption caused by the O^{2-}→Yb^{3+} charge transfer (CT) transition.

Part 3. Crystal structure parameters of the studied $Y_{1-x}Gd_xAlO_3:Mn^{4+}$ (x=0, 0.2, 0.4, 0.6, 0.8, 1) and $Gd_{1-y}La_yAlO_3:Mn^{4+}$ (y=0.2, 0.3, 0.4) phosphors

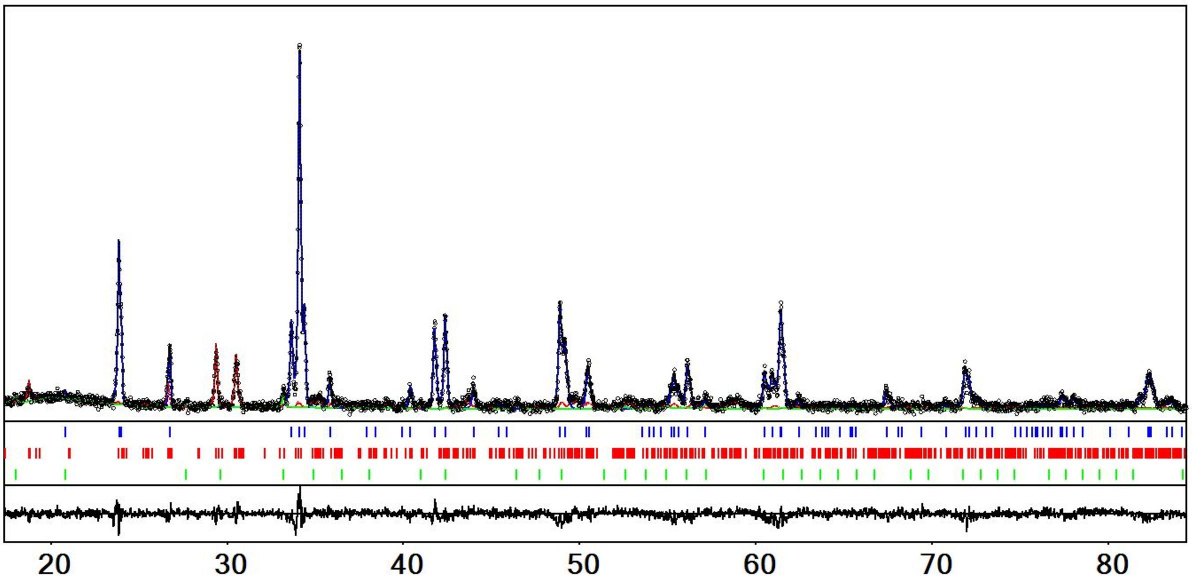

Figure S5. Graphical results of Rietveld refinement showing coexistence of the perovskite (blue), monoclinic (red) and garnet (green) phases in the $Y_{0.6}Gd_{0.4}AlO_3:Mn^{4+}$ sample. Experimental XRD data (black circles) are shown in comparison with calculated patterns for each phase. Short vertical bars indicate positions of Bragg's maxima in corresponding structures.

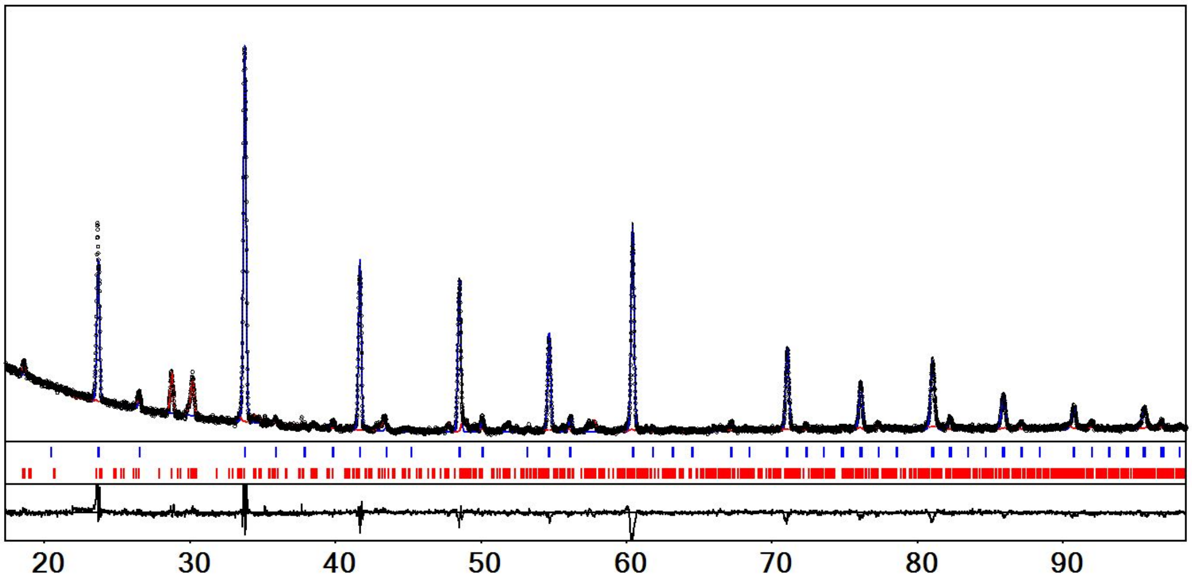

Figure S6. Graphical results of Rietveld refinement showing coexistence of the perovskite (blue) and monoclinic (red) phases in the $Gd_{0.7}La_{0.3}AlO_3:Mn^{4+}$ sample. Experimental XRD data (black circles) are shown in comparison with calculated patterns for both phases. Short vertical bars indicate positions of Bragg's maxima in corresponding structures.

Table S4. Refined lattice parameters (*a*, *b*, *c*) and unit cell volume (*V*) of $Y_{1-x}Gd_xAlO_3:Mn^{4+}$ (x=0, 0.2, 0.4, 0.6, 0.8, 1) and $Gd_{1-y}La_yAlO_3:Mn^{4+}$ (y=0.2, 0.3, 0.4) perovskites.

x, y	*a*, Å	*b*, Å	*c*, Å	*V*, Å^3
$Y_{1-x}Gd_xAlO_3:Mn^{4+}$				
0	5.1789(2)	5.3286(2)	7.3699(3)	203.38(2)
0.2	5.1968(3)	5.3231(2)	7.3898(3)	204.42(3)
0.4	5.2103(4)	5.3201(4)	7.4042(5)	205.24(4)
0.6	5.2239(4)	5.3133(4)	7.4179(6)	205.89(5)
0.8	5.2429(5)	5.3053(4)	7.4367(7)	206.85(5)
1	5.2452(6)	5.3040(6)	7.4386(9)	206.95(7)
$Gd_{1-y}La_yAlO_3:Mn^{4+}$				
0.2	5.2918(3)	5.2999(3)	7.4790(4)	209.75(3)
0.3	5.3048(5)	5.3017(3)	7.4920(5)	210.71(5)
0.4	5.3212(2)	5.3054(2)	7.4999(3)	211.73(2)

Part 4. Estimation of trap depths from TSL

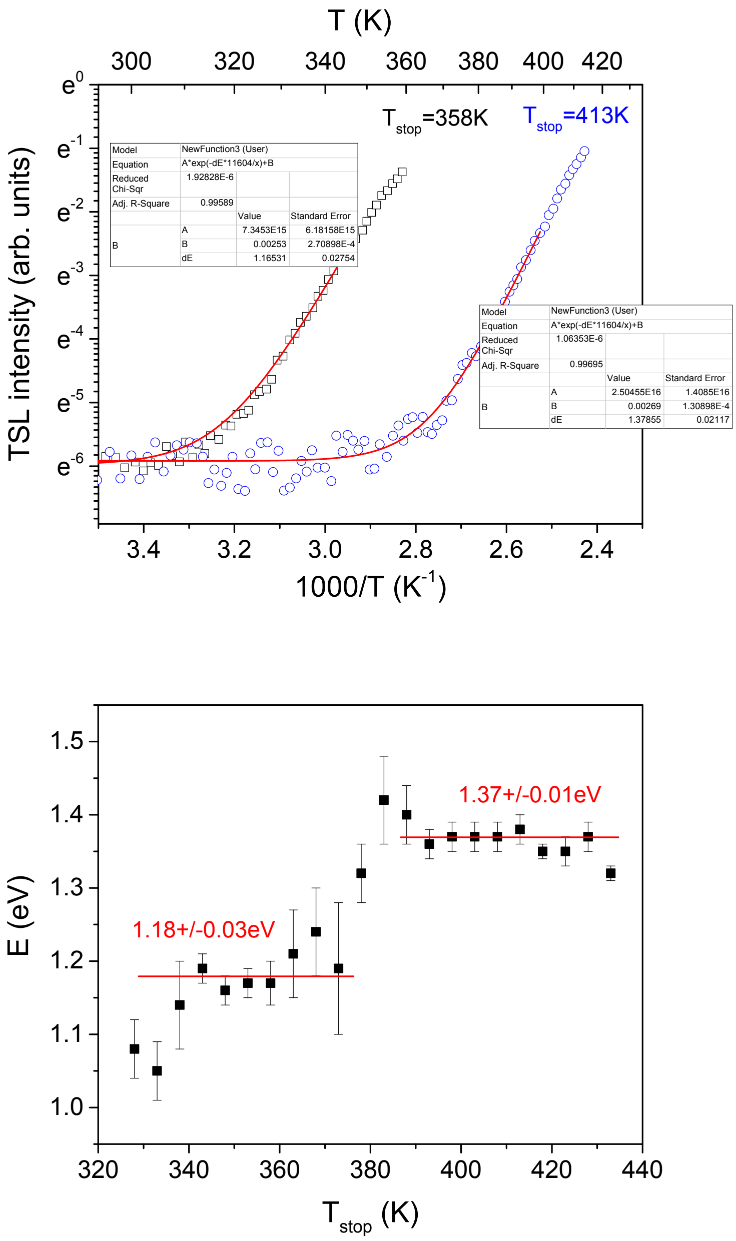

Figure S7. Partial results demonstrating the trap depths estimated by the initial rise method in the partial cleaning procedure applied for the $Y_{0.2}Gd_{0.8}AlO_3:Mn^{4+}$ sample.

Part 5. Calculated energy levels and trap depths of several defects within the band gap of $YAlO_3$

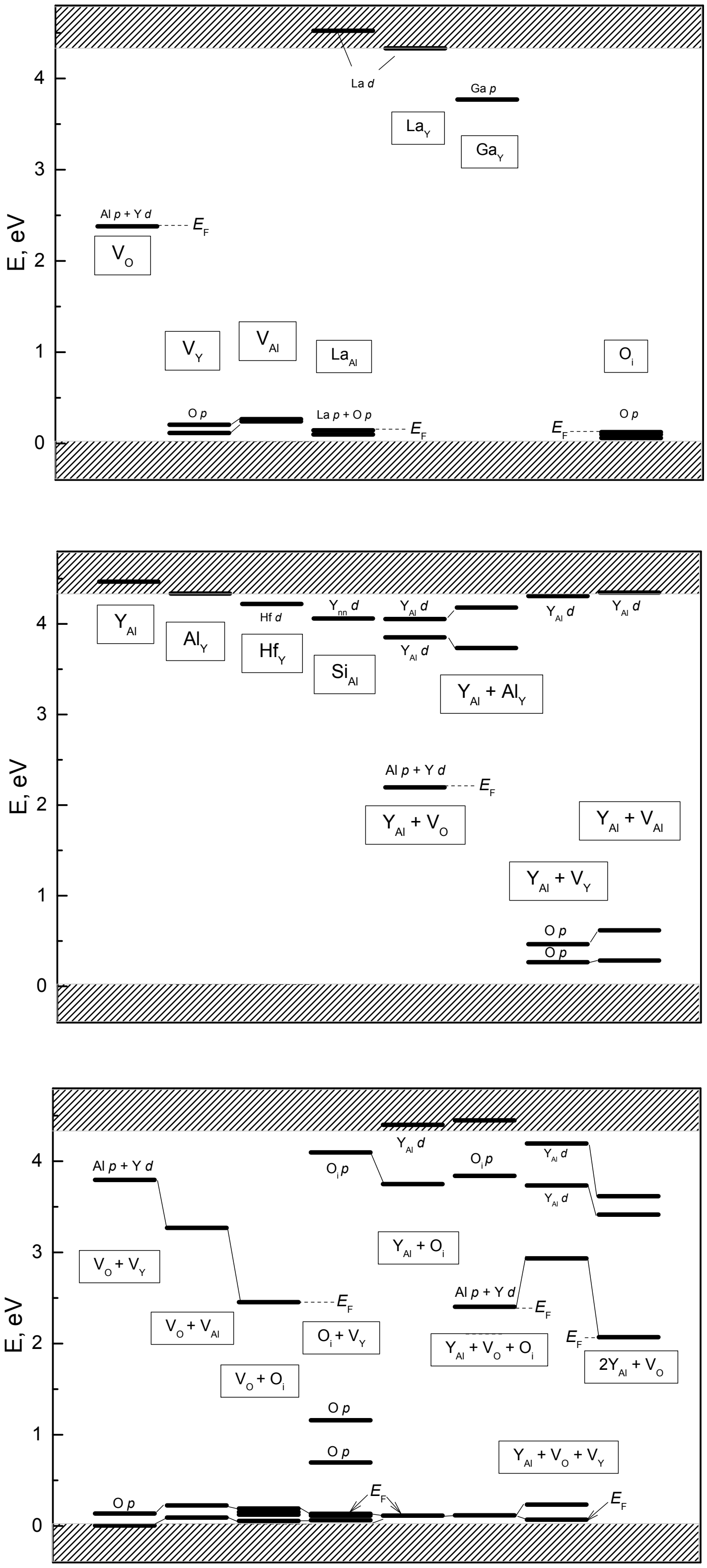

Figure S8. Calculated energy levels of several defects within the band gap of a $YAlO_3$ crystal (atomic orbital characters for the defect levels and calculated Fermi energies E_F are indicated, E_F = 0 if not indicated).

Table S5. Calculated trap depths (in eV) of native, interstitial, substitution and antisite defects in $YAlO_3$ (Δ(α-β) denotes the difference in trap depths between spin polarizations obtained in calculations).

Defect	h-trap	e-trap	Δ(α-β)	Defect	h-trap	e-trap	Δ(α-β)
V_O		2.38	$< 10^{-3}$	$Y_{Al} + V_O$	2.196	0.32<br>0.522<br>2.178	$< 10^{-3}$
V_Y	0.116<br>0.206		0.05	$Y_{Al} + V_Y$	0.266<br>0.465	0.047	0.3
V_{Al}	0.242<br>0.267		0.05	$Y_{Al} + V_{Al}$	0.033<br>0.285<br>0.619		0.3
O_i	0.06<br>0.099<br>0.125		$< 10^{-3}$	$Y_{Al} + O_i$	0.113	0.65	$< 10^{-3}$
Hf_Y		0.124	0.05	$Y_{Al} + V_O + O_i$	0.117<br>2.404	0.474<br>1.910	$< 10^{-3}$
Si_{Al}		0.277	$< 10^{-3}$	$Y_{Al} + V_O + V_Y$	0.07<br>0.234	0.146<br>0.606<br>1.407	<0.1
Y_{Al}			$< 10^{-3}$	$Y_{Al} + V_O + V_Y$<br>in $Y_{0.75}La_{0.25}AlO_3$	0.033<br>0.172	0.105<br>0.454<br>1.238	<0.1
Al_Y		0.013	$< 10^{-3}$	$V_O + V_Y + 2Y_{Al}$	0.026<br>0.101<br>1.108	0.12<br>0.626<br>1.015	<0.1
$V_O + V_Y$	0.005<br>0.137	0.467	$< 10^{-3}$	$Y_{Al} + Al_Y$		0.184<br>0.631	$< 10^{-3}$
$V_O + V_{Al}$	0.092<br>0.226	0.925	0.15	$2Y_{Al} + V_O$	2.071	0.711<br>0.913	
$V_O + O_i$	0.056<br>0.125<br>0.154<br>0.191<br>2.454	1.864	$< 10^{-3}$	La_Y		0.009	$< 10^{-3}$
$V_Y + O_i$	0.063<br>0.113<br>0.133<br>0.697<br>1.159	0.215	0.3	La_{Al}	0.098<br>0.144		$< 10^{-3}$
				Ga_Y		0.607	
